# MRI-scale histology validates spatial sensitivity of in-vivo MRI-based axon radius estimation

**DOI:** 10.1162/IMAG.a.1030

**Published:** 2025-12-03

**Authors:** Laurin Mordhorst, Luke J. Edwards, Maria Morozova, Mohammad Ashtarayeh, Tobias Streubel, Björn Fricke, Francisco J. Fritz, Henriette Rusch, Carsten Jäger, Ludger Starke, Thomas Gladytz, Ehsan Tasbihi, Joao S. Periquito, Andreas Pohlmann, Herbert Mushumba, Klaus Püschel, Thoralf Niendorf, Nikolaus Weiskopf, Markus Morawski, Siawoosh Mohammadi

**Affiliations:** Department of Neuroradiology, University of Luebeck, Luebeck, Germany; Institute of Systems Neuroscience, University Medical Center Hamburg-Eppendorf, Hamburg, Germany; Department of Cognitive Neuroscience, Faculty of Psychology and Neuroscience, Maastricht University, Maastricht, The Netherlands; Department of Neurophysics, Max Planck Institute for Human Cognitive and Brain Sciences, Leipzig, Germany; Paul Flechsig Institute—Centre of Neuropathology and Brain Research, Leipzig, Germany; Berlin Ultrahigh Field Facility (B.U.F.F.), Max Delbrück Center for Molecular Medicine in the Helmholtz Association, Berlin, Germany; Charité—Universitätsmedizin Berlin, Berlin, Germany; Institute of Legal Medicine, University Medical Center Hamburg-Eppendorf, Hamburg, Germany; Felix Bloch Institute for Solid State Physics, Faculty of Physics and Earth System Sciences, Leipzig University, Leipzig, Germany; Wellcome Centre for Human Neuroimaging, UCL Queen Square Institute of Neurology, University College London, London, UK; Max Planck Research Group MRI Physics, Max Planck Institute for Human Development, Berlin, Germany

**Keywords:** axon radius, microstructure, diffusion-weighted MRI, light microscopy, histology, validation

## Abstract

The axon radius holds promise as a clinical MRI biomarker for neurological disorders. However, in-vivo MRI estimation appears infeasible on clinical scanners and lacks experimental validation. Crucially, existing histology is only sparsely sampled, enabling primarily qualitative assessment. Here, we use large-scale human brain histology, sampling 46 million axons across 35 corpus callosum regions with MRI-like sizes. By demonstrating a significant spatial correlation with histology on an advanced research scanner, we provide quantitative proof that MRI radius estimates reflect underlying microstructure—a critical milestone. The next milestone—translation to clinical scanners—appears feasible with now-available high-gradient systems according to simulations, but would require substantial SNR gains. Yet, we also identify a sensitivity bottleneck in current modeling that may offer a complementary path to improved sensitivity through future modeling advances. Overall, we provide promising evidence for the validity of MRI-based axon radius estimation and identify challenges that must be solved for clinical adoption.

## 1 Introduction

Axons are critical to neural communication, with their radii influencing communication speed (Waxman, 1980). Axon radii vary spatially along and across white matter fiber bundles (Caminiti et al., 2009; Tomasi et al., 2012; Veraart et al., 2021) but also change temporally over the lifespan. While typical changes occur during development and aging, others can indicate neurodevelopmental disorders (Stassart et al., 2018; Wegiel et al., 2018) or neurodegenerative diseases (Evangelou et al., 2001; Judson et al., 2017), positioning the axon radius as a potential clinical biomarker.

This biomarker might be measurable via water diffusion-weighted magnetic resonance imaging (dMRI). In the dMRI-based signal models relevant here, axons are represented as cylindrical structures, with diffusion perpendicular to the cylinder axes reflecting the axon radius (Alexander et al., 2010; Assaf et al., 2008; Sepehrband, Alexander, Kurniawan, et al., 2016; Veraart et al., 2020). Since axon radii are micrometer-sized—much smaller than millimeter-scale dMRI voxels—the dMRI signal reflects a combined contribution from the individual axons within a voxel. This combined contribution of all axons within a voxel has been proposed to be captured in a scalar metric, the effective axon radius ($r_{\text{eff}}$ ) (Burcaw et al., 2015; Veraart et al., 2020), which is heavily influenced by the largest axons, representing the tail of the axon radius distribution.

Estimating $r_{\text{eff}}$ in real-world dMRI experiments, and axon radius metrics more broadly, has proven challenging throughout the past decades, mainly due to dMRI’s weak sensitivity to small axon radii (Huang et al., 2015; Nilsson et al., 2017; Sepehrband, Alexander, Kurniawan, et al., 2016; Veraart et al., 2020). In-vivo, the gradient amplitudes required to exploit this sensitivity have only recently become accessible with the advent of a handful of specialized research scanners reaching up to 300 mT​/​m (Fan et al., 2020; Pizzolato et al., 2023; Veldmann et al., 2024; Veraart et al., 2020, 2021). While current clinical systems are limited to gradient amplitudes up to 80 mT​/​m , next-generation scanners reaching up to 200 mT​/​m may help bridge this gap and open new possibilities for clinical translation. In parallel with these hardware advances, the understanding of dMRI signal contributions has evolved. One line of work revisits the core intra-axonal signal model, using simulations of at most a few hundred axons (M. Andersson et al., 2020, 2022; H.-H. Lee, Papaioannou, et al., 2020; H.-H. Lee et al., 2019, 2024; Winther et al., 2024) to explore how complex axonal morphology alters the dMRI signal relative to the simplified cylinder assumption underlying $r_{\text{eff}}$ . Another line of research focuses on confounding signal contributions from outside the axon, such as axonal surface relaxation effects (Barakovic et al., 2023), additional signal compartments (Alexander et al., 2010; Palombo et al., 2020; Pizzolato et al., 2023; Stanisz et al., 1997; Veraart et al., 2019, 2020), orientation dispersion (Drobnjak et al., 2016; Nilsson et al., 2012), and Rician noise bias (Gudbjartsson & Patz, 1995), with various strategies proposed to address these confounding signal contributions via experimental design (Veraart et al., 2020, 2021), modeling (Barakovic et al., 2023; Jespersen et al., 2013; Kaden et al., 2016; Mollink et al., 2017; Pizzolato et al., 2023), and processing (Varadarajan & Haldar, 2015).

Despite these challenges, spatial trends in $r_{\text{eff}}$ have emerged with some consistency, particularly in the corpus callosum (Fan et al., 2019; Horowitz, Barazany, Tavor, Bernstein, et al., 2015; Pizzolato et al., 2023; Veraart et al., 2021). In rats, a low-high-low profile has been reported (Barazany et al., 2009), aligning well with histological findings (Barazany et al., 2009; Veraart et al., 2020). However, similar patterns in humans (Aboitiz et al., 1992; Barakovic et al., 2021, 2023; Caminiti et al., 2009; Horowitz, Barazany, Tavor, Bernstein, et al., 2015; Huang et al., 2015; Pizzolato et al., 2023; Veraart et al., 2021) and nonhuman primates (Caminiti et al., 2009; Lamantia & Rakic, 1990) appear less consistent. Critically, these dMRI-histology comparisons have remained largely qualitative rather than quantitative—mainly due to limitations in current histological datasets, particularly for humans. These comparisons rely on 2D histology, which attempts to sample axon radius distributions orthogonal to the fiber direction in highly aligned white matter such as the corpus callosum (Aboitiz et al., 1992; Barakovic et al., 2023; Caminiti et al., 2009; Liewald et al., 2014; Veraart et al., 2020). However, in humans, these datasets typically include only a small number of regions of interest (ROIs) (Aboitiz et al., 1992; Barakovic et al., 2023; Caminiti et al., 2009; Liewald et al., 2014), precluding meaningful spatial correlation analysis. Moreover, the ROIs often cover only a fraction of a dMRI voxel, which—due to the tail-weighting of $r_{\text{eff}}$ —makes estimates prone to strong statistical fluctuations (Barakovic et al., 2023; Veraart et al., 2020). In rats, broader spatial sampling has been attempted, including 20 ex-vivo dMRI voxel-sized ROIs in the corpus callosum (Veraart et al., 2020). Yet, the variation in $r_{\text{eff}}$ across these ROIs is minimal, and the analysis largely focuses on distribution-level agreement rather than ROI-wise correspondence, limiting conclusions about spatial sensitivity and potentially masking systematic error, for example, through compensating contributions of confounding factors.

Recently, 3D histology has emerged as a complementary approach (M. Andersson et al., 2020; H.-H. Lee et al., 2019; Shapson-Coe et al., 2024; Tian et al., 2025), though only few studying human tissue (Shapson-Coe et al., 2024; Tian et al., 2025). These data reveal complex axonal morphology, including undulations and radius fluctuations, inaccessible to 2D histology. However, current 3D datasets cover at most three ROIs in animals (M. Andersson et al., 2020; H.-H. Lee et al., 2019)—or even just a single ROI in humans (Shapson-Coe et al., 2024; Tian et al., 2025)—and have largely been used to simulate morphological effects on dMRI signal formation, rather than for direct experimental validation.

In this study, we aim to provide the first quantitative proof of sensitivity of dMRI-based $r_{\text{eff}}$ measurements to underlying tissue microstructure. To this end, we assess quantitative spatial correlations between $r_{\text{eff}}$ from experimental in-vivo and ex-vivo dMRI against corresponding histological values, enabled by a densely sampled light microscopy dataset comprising 35 in-vivo dMRI voxel-sized ROIs across two human corpora callosa. First, we establish our light microscopy-based histology dataset as a benchmark for experimental $r_{\text{eff}}$ validation by demonstrating gains in accuracy and precision over existing datasets (Aboitiz et al., 1992; Barakovic et al., 2023; Caminiti et al., 2009; Liewald et al., 2014). We then show that $r_{\text{eff}}$ estimates from in-vivo dMRI significantly correlate with histological values, despite a newly identified model-inherent sensitivity reduction in the dMRI-based *r*eff. Finally, we show that next-generation clinical scanners—now available on the market—may bring in-vivo $r_{\text{eff}}$ mapping within the realm of clinical adoption, although substantial improvements in SNR remain necessary.

## 2 Theory: The dMRI-visible Effective Axon Radius ${\text{r}}_{\text{eff}}$

Here, we provide a brief introduction to the dMRI-visible effective axon radius $r_{\text{eff}}$ and the dMRI signal model used for its estimation. For a more detailed description, see Appendix A.1. The dMRI-visible effective axon radius

$$r_{\text{eff}}=\sqrt[{4}]{\frac{\langle r^{6}\rangle }{\langle r^{2}\rangle }}.$$ (1)

is a scalar, tail-weighted statistic of the axon radius distribution within a dMRI voxel (Burcaw et al., 2015; Veraart et al., 2020). $r_{\text{eff}}$ can be estimated in a regime of strong diffusion weighting (b), suggested to be around $b\;\geq \;\;{\text{6 ms/µm}}^{2}$ for in-vivo dMRI (Veraart et al., 2019, 2020) and $b\geq {\text{20 ms/µm}}^{2}$ for ex-vivo dMRI (Veraart et al., 2020). In this regime, one can approximate the powder-averaged dMRI signal as

$$S^{\circ }(b)\approx \frac{\beta }{\sqrt{b}}\cdot e^{-bD_{\text{a}}^{⊥}}+f_{\text{im}},$$ (2)

where β is a signal scaling factor, $D_{\text{a}}^{⊥}$ is the intra-axonal perpendicular diffusivity, and $f_{\text{im}}$ is the signal of the immobile water compartment (Alexander et al., 2010; Stanisz et al., 1997). $r_{\text{eff}}$ is directly linked to $D_{\text{a}}^{⊥}$ through

$$r_{\text{eff}}=\sqrt[{4}]{\frac{48}{7}\delta \left(\Delta -\frac{\delta }{3}\right)D_{\text{a}}^{⊥}D_{0}},$$ (3)

where δ is the diffusion gradient duration, Δ is the diffusion gradient separation, and $D_{0}$ is the diffusivity of the axoplasm (Veraart et al., 2020). Using Equations (2) and (3) and an estimate for $D_{0}$, one can jointly estimate $r_{\text{eff}}$ and β, for example, via non-linear fitting (Veraart & Novikov, 2019).

## 3 Materials and Methods

### 3.1 Histology

#### 3.1.1 Tissue samples

We used two human corpus callosum tissue samples, CC-01 and CC-02. See Table 1 for sample information.

**Table 1.** Tissue sample information for the ex-vivo dataset.

Sample	Sex	Age [years]	Postmortem delay [hours]	Cause of death	Histology ROIs	Ex-vivo dMRI ROIs
CC-01	m	61	20	myocardial infection	16	15
CC-02	f	60	24	multi organ failure	19	0
					35	15

**Fig. 1.**
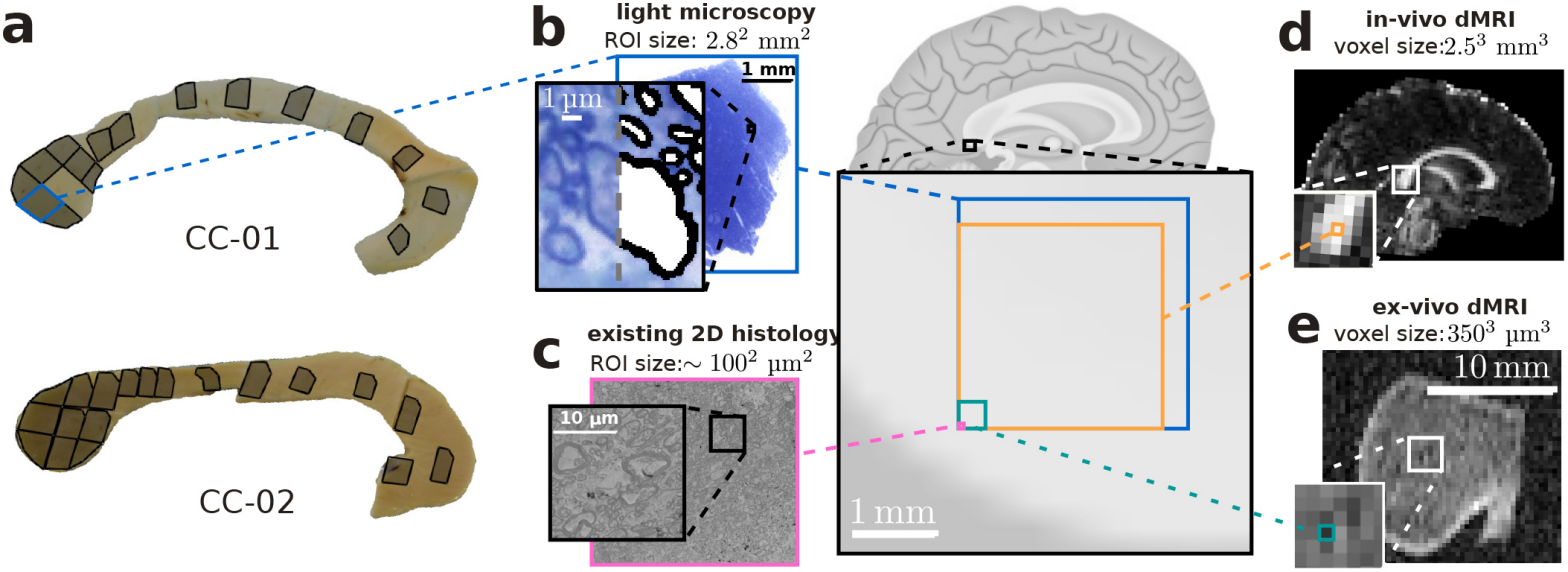
An in-vivo dMRI-scale histological reference for $r_{\text{eff}}$ . (a) Human corpus callosum tissue samples with annotated regions of interest (ROIs) imaged via light microscopy. Note that the dense splenium sampling reflects the design of a companion study focused on the posterior callosal area. (b–e) Contextualization of light microscopy ROI size relative to other modalities. The central image shows a mid-sagittal sketch of the human brain with a magnified view of a corpus callosum subregion, illustrating ROI and voxel sizes for (b) light microscopy, showing one ROI from (a) with a magnified view illustrating myelin sheath (black) and axonal body (white) segmentation; (c) electron microscopy, using an image from Mordhorst et al. (2022), with an ROI size representative of existing 2D histology datasets of the human corpus callosum (Aboitiz et al., 1992; Barakovic et al., 2023; Caminiti et al., 2009; Liewald et al., 2014); (d) in-vivo dMRI from this study, shown as a fractional anisotropy map of an exemplary subject; (e) ex-vivo dMRI from this study, shown as a diffusion-weighted image.

**Fig. 2.**
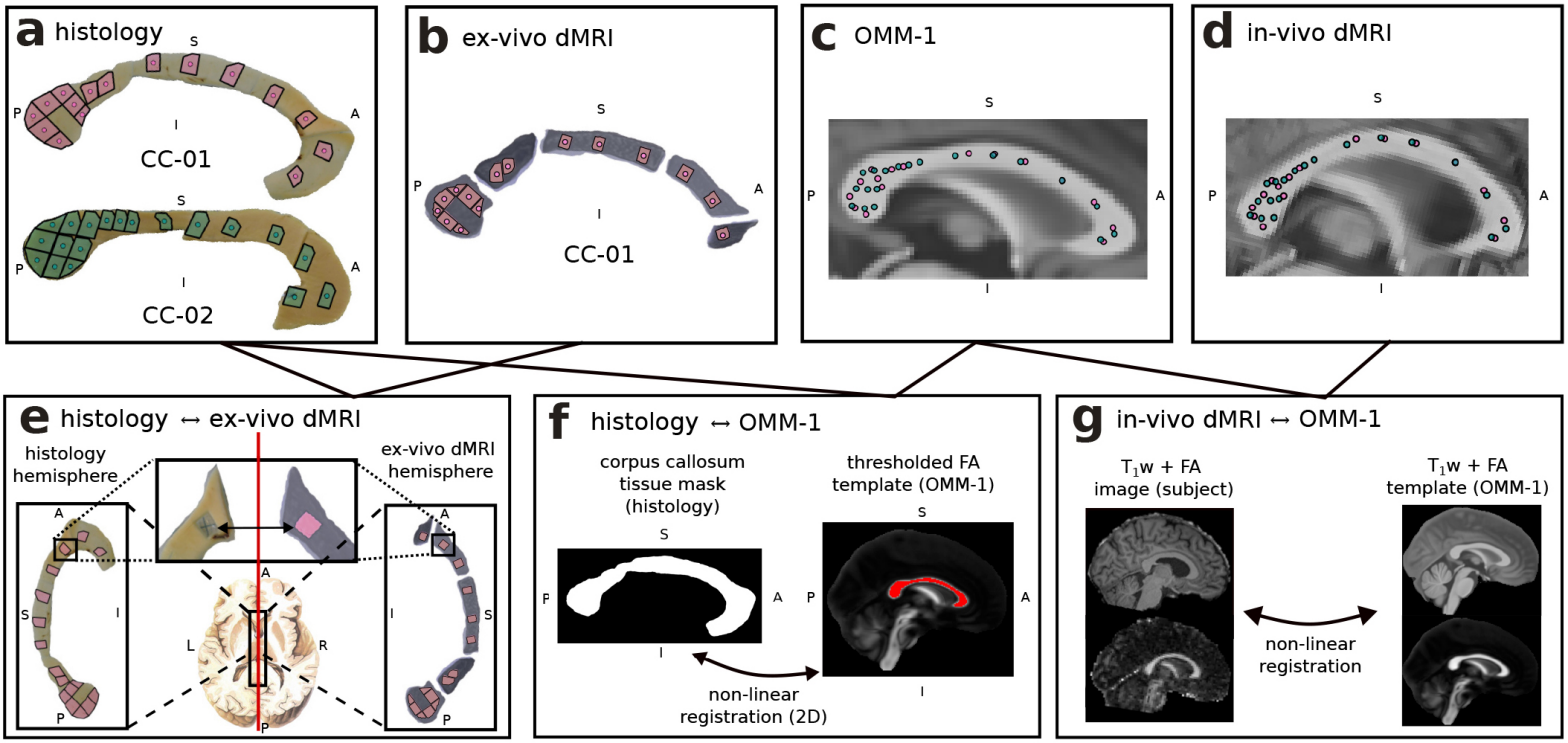
Regions of interest in different spaces and their registration. (a–d) Regions of interest (ROIs) shown in different spaces: (a) histology, (b) ex-vivo dMRI, (c) Oxford-MultiModal-1 (OMM-1, Arthofer et al., 2024) atlas space (overlaid on T1-weighted template), (d) in-vivo dMRI (overlaid on T1-weighted image). Polygons and circles indicate ROI boundaries and centroids, with colors representing tissue sample CC-01 (magenta) or CC-02 (green). (e) Registration between histology and ex-vivo dMRI. We bisected the brain along the mid-sagittal plane, indicated by the red line, yielding hemispheric sections for histology (left) and ex-vivo dMRI (right). We first defined histological ROIs near the mid-sagittal plane; then, we manually defined corresponding ROIs in ex-vivo dMRI. Magnified views illustrate an example of matching ROIs in histology and ex-vivo dMRI (extracted tissue area in histology and magenta area in ex-vivo dMRI). Note that we scanned only part of the genu with ex-vivo dMRI. (f) Registration between histology and OMM-1 space. We manually created two-dimensional tissue masks (left image) for the images in (a) and registered these masks with the mid-sagittal slice of the OMM-1 fractional anisotropy (FA) atlas (thresholded at FA≥0.6 , see red area in right image). (g) Registration between OMM-1 space and in-vivo dMRI. We simultaneously registered $T_{1}$-weighted image and FA map in native space to their corresponding templates in OMM-1 space.

*Histology ROIs* and *Ex-vivo dMRI ROIs* indicate the number of regions of interest (ROIs) analyzed in each modality. See Figure 1a for locations of all histology ROIs and Figure 2a, b, e for common ROIs between histology and ex-vivo dMRI.

#### 3.1.2 Tissue preparation

We immersion-fixed whole brains in 3%. paraformaldehyde and 1% glutaraldehyde in phosphate-buffered saline (pH 7.4). We then extracted the corpora callosa and bisected them along the mid-sagittal plane, yielding hemispheric sections. We prepared one hemispheric section from each donor for histology and preserved the other hemisphere for ex-vivo dMRI. In the histology hemisphere, we cut a slice of the tissue sample orthogonal to the mid-sagittal plane and extracted 35 ROIs in total, each roughly including the cross-sectional area of in-vivo dMRI voxels used in our study (see Fig. 1). The ROI segments were then contrasted with osmium tetroxide and uranyl acetate, dehydrated in graded acetones, and embedded in Durcupan resin. For imaging with light microscopy, we cut semi-thin sections (≈500 nm thickness) parallel to the mid-sagittal plane on a Reichert Ultractut II. The sections were mounted on Thermo Scientific SuperFrost Plus glass slides, stained with 1% toluidine blue, air dried, and coverslipped with Sigma-Aldrich Entellan toluene.

#### 3.1.3 Image acquisition

We acquired light microscopy images (one per ROI) using a Zeiss AxioScan Z1 (objective: 40× , numerical aperture: 0.95, resolution: 0.1112  µm /pixel; resolution limit: 292 nm ). An example image is shown in Figure 1b.

#### 3.1.4 Parameter estimation

We segmented axons using a deep-learning-based method (Mordhorst et al., 2022) (see Fig. 1c) and derived empirical axon radius distributions by calculating the radius of circles with equivalent areas for each axon. For comparison with in-vivo dMRI, we compensated for tissue shrinkage by scaling each axon radius (scaled radii: $r^{\prime }=1.3r$ ), where the factor 1.3 was estimated as the mean of previously reported values (Aboitiz et al., 1992; Tang et al., 1997). Finally, we computed $r_{\text{eff}}$ values from the empirical axon radius distributions using Equation (1).

### 3.2 Ex-vivo dMRI

#### 3.2.1 Tissue samples

For ex-vivo dMRI, we used the hemisphere of CC-01 that was not processed for histology (see Fig. 2e for the bisection into hemispheres). Sample information is provided in Table 1.

#### 3.2.2 Tissue preparation

We cut the tissue sample into five segments along the anterior-posterior axis (see Fig. 2b) using a Reichert Ultracut II, and embedded the segments in 1.5% agarose in phosphate buffer saline in a custom-made container.

#### 3.2.3 Image acquisition

We acquired magnitude dMRI data using a Bruker Biospin 9.4T scanner with a single-channel transceiver volume coil and a gradient insert coil with a maximum gradient amplitude of 1500 mT​/​m at the Berlin Ultrahigh Field Facility in Berlin, Germany. We followed a protocol similar to that suggested by Veraart et al. (2020) for ex-vivo $r_{\text{eff}}$ mapping in rats. Briefly, we applied diffusion-weighting for 65 gradient directions per b, with directions based on the approach by Dubois et al. (2006). We used a segmented EPI sequence with four segments and the following fixed parameters: δ=7 ms , Δ=20.1 ms , echo time $T_{\text{E}}=\text{34.7 ms}$ , repetition time $T_{\text{R}}=[15000,\;25000]\;\;\text{ms}$ (segment-dependent), and isotropic voxel edge length of 0.35 mm . For different tissue segments, the field-of-view was adjusted between $22\;\times \;28\;\times \;{\text{9 mm}}^{3}$ and 25 × 30 ×  ${\text{10.5 mm}}^{3}$. We varied b between 2.5 and $100\text{ ms}/{\text{µm}}^{2}$, and gradient amplitude (g) between 200 and 1278 mT​/​m , as detailed in Table 2. To enhance SNR, we averaged repeated measurements prior to image reconstruction for higher b-values, as shown in the *Repetitions* column of Table 2.

**Table 2.** Ex-vivo dMRI acquisition parameters.

b $\left[{\text{ms/µm}}^{2}\right]$	g [mT/m]	Repetitions
2.5	200	1
5.0	283	1
7.5	347	1
10.0	401	1
20.0	567	2
30.0	695	2
40.0	802	3
50.0	896	3
60.0	982	4
70.0	1061	5
80.0	1134	6
90.0	1203	7
100.0	1278	8

*Repetitions* denotes the number of repeated measurements per diffusion gradient direction, which were averaged in k-space prior to image reconstruction.

#### 3.2.4 Preprocessing

We corrected for Gibbs ringing artifacts (Kellner et al., 2016; Tournier et al., 2019). To account for signal drift across b-shells, we normalized images within each b-shell to an S(b=0) image acquired at the start of acquisition for that shell.

#### 3.2.5 Parameter estimation

Per b, we estimated the noise level $\hat{\sigma }$ using Marchenko-Pastur principal component analysis (Cordero-Grande et al., 2019; Tournier et al., 2019; Veraart et al., 2016) prior to preprocessing. Then, we estimated $S^{\circ }(b)$ as the zeroth-order spherical harmonic using an estimator of the even order spherical harmonic coefficients up to the sixth order. Specifically, we determined the spherical harmonics basis functions (Coelho et al., 2022, 2023; Novikov et al., 2018; Reisert et al., 2017) and estimated the coefficients using a Rician maximum likelihood estimator (Varadarajan & Haldar, 2015), which relied on the b-dependent $\hat{\sigma }$ maps.

For $b\leq {\text{10 ms/µm}}^{2}$, we estimated the main fiber direction $\vec{\mu }$ using NODDI (Zhang et al., 2012a, 2012b), using the $\hat{\sigma }$ map for $b={\text{2.5 ms/µm}}^{2}$.

For $b\geq {\text{20 ms/µm}}^{2}$, we estimated $r_{\text{eff}}$ from $S^{\circ }(b)$ using Equations (2) and (3) via non-linear fitting (Veraart & Novikov, 2019), assuming $D_{0}\;\;={\text{0.35 µm}}^{\text{2}}\text{/ms}$ (West et al., 2018). We estimated $f_{\text{im}}$ (see Equation (2)) from strongly decayed directional signals. To this end, we selected signals from the highest b-shell with high alignment between $\vec{g}$ and $\vec{\mu }$ (angle ≤20° ), fitted a Rician distribution, and approximated $f_{\text{im}}$ as its expected value.

### 3.3 In-vivo dMRI

#### 3.3.1 Subjects

We recruited five healthy adult subjects (age: 31  ±3 years, representing mean ± standard deviation; sex: 2 male, 3 female).

#### 3.3.2 Image acquisition

We acquired magnitude dMRI data using a 32-channel receive coil and 300 mT​/​m gradient coils on a Siemens Connectom 3T scanner at the Max Planck Institute for Human Cognitive and Brain Sciences in Leipzig, Germany. We followed the dMRI protocol described by Veraart et al. (2021). Briefly, we used a single-shot multi-band echo-planar imaging (EPI) sequence with blipped-CAIPI (multi-band factor: 2) and in-plane GRAPPA acceleration (acceleration factor: 2). We applied diffusion-weighting with the following fixed parameters: δ=15 ms , Δ =30 ms , $T_{\text{E}}=\text{66 ms}$ , $T_{\text{R}}=\text{3500 ms}$ , matrix size of 88× 88 with 54 slices, and isotropic voxel edge length of 2.5 mm . We varied $b=\{0.5,\;1,\;2.5,\;6,\;30.45\}\;{\text{ms/µm}}^{2}$ for {30, 30, 30, 60, 120} gradient directions isotropically distributed on a sphere (Jones et al., 1999) and used variable gradient amplitude g={36, 51, 80, 124, 279} mT/m .

For geometric susceptibility correction, we acquired 23 non-diffusion-weighted images with the same and 10 images with reverse phase encoding. Additionally, we acquired T1-weighted MP-RAGE images (Brant-Zawadzki et al., 1992).

#### 3.3.3 Preprocessing

We corrected for Gibbs ringing artifacts (Kellner et al., 2016; Tournier et al., 2019), eddy current and motion artifacts (J. L. R. Andersson & Sotiropoulos, 2016; J. L. R. Andersson et al., 2016; Tournier et al., 2019), and gradient non-linearity distortions (Janke et al., 2004; Jovicich et al., 2006).

#### 3.3.4 Parameter estimation

For $b\;\;\leq {\text{2.5 ms/µm}}^{2}$, we estimated the noise level $\hat{\sigma }$ using Marchenko-Pastur principal component analysis (Cordero-Grande et al., 2019; Tournier et al., 2019; Veraart et al., 2016) prior to preprocessing. After preprocessing, we estimated the apparent diffusion tensor (Basser et al., 1994a, 1994b; Tournier et al., 2019) and mapped fractional anisotropy (FA) (Basser et al., 1994a; Tournier et al., 2019).

For $b\geq {\text{6 ms/µm}}^{2}$, we estimated $S^{\circ }(b)$ as the zeroth-order spherical harmonic using an estimator of the even order spherical harmonic coefficients up to the sixth order. Specifically, we determined the spherical harmonics basis functions (Coelho et al., 2022, 2023; Novikov et al., 2018; Reisert et al., 2017) and estimated the coefficients using a Rician maximum likelihood estimator (Varadarajan & Haldar, 2015), which relied on the $\hat{\sigma }$ maps. Finally, we estimated $r_{\text{eff}}$ using Equations (3) and (A14), assuming $D_{0}={\text{2.07 µm}}^{\text{2}}\text{/ms}$ (Veraart et al., 2018) and $f_{\text{im}}=0$ (Tax et al., 2020).

### 3.4 dMRI simulations

We conducted simulations to replicate dMRI signal generation and $r_{\text{eff}}$ -estimation under ex-vivo and in-vivo conditions. The simulations are described in greater detail in Appendix A.2. In brief, we generated dMRI signals for each diffusion gradient direction by computing volume-weighted average signals (Packer & Rees, 1972) over our empirical axon radius distributions. For in-vivo simulations, we used axon radius distributions adjusted for tissue shrinkage as described in the histology section. For signal simulation, we modeled three compartments: intra-axonal, extra-axonal, and immobile water compartment with $T_{2}$-weighted volume fractions $f_{\text{a}}$, $f_{\text{e}}$ and $f_{\text{im}}$ . We simulated intra-axonal signal using the matrix method (Callaghan, 1997) to capture effects beyond the Gaussian phase approximation (Van Gelderen et al., 1994). We assumed fully decayed extra-axonal signal (but $f_{\text{e}}\;\;>0$ ) and used fixed $f_{\text{im}}$ . To estimate $r_{\text{eff}}$ from simulated signals, we followed the procedure for experimental ex-vivo and in-vivo dMRI data, assuming known $f_{\text{im}}$ . We considered both an idealized scenario (SNR=∞ ), as well as an “experiment-like” scenario mimicking experimental Rician noise conditions (in-vivo: SNR = 32; ex-vivo: b-dependent SNR ranging from 17 to 51), for which we repeated simulations 1000 times.

### 3.5 Quantification of improvements through dMRI-scale histology

For each histology ROI, we empirically determined the sampling distribution of $r_{\text{eff}}$ for different subsample sizes between $10^{2}$ and $10^{5}$ axons, reflecting smaller ROI sizes typical for existing histology data (Aboitiz et al., 1992; Caminiti et al., 2009; Liewald et al., 2014). Per subsample size, we assessed accuracy using the normalized mean bias error

$$\text{NMBE}=\frac{\sum_{m=1}^{M}({\hat{r}}_{\text{eff},m}-r_{\text{eff}})}{r_{\text{eff}}}$$ (4)

and precision using the coefficient of variation

$$\text{CV}=\frac{\text{std}(\{{\hat{r}}_{\text{eff},\text{m}}|m\in M\})}{r_{\text{eff}}},$$ (5)

where M=1000 , ${\hat{r}}_{\text{eff},\text{m}}$ is a subsample estimate, $r_{\text{eff}}$ is the reference value computed from the full empirical axon radius distribution, and $\text{std}(\{{\hat{r}}_{\text{eff},\text{m}}|m\in M\})$ denotes the standard deviation across all subsample estimates. This subsampling analysis assumes zero spatial autocorrelation of axon radii.

### 3.6 Comparison of ${\text{r}}_{\text{eff}}$ across modalities

#### 3.6.1 Qualitative comparison of spatial ${\text{r}}_{\text{eff}}$ patterns

To compare the spatial patterns of $r_{\text{eff}}$ in the mid-sagittal section of the corpus callosum across modalities, we mapped $r_{\text{eff}}$ values from all modalities onto the mid-sagittal slice in Oxford-MultiModal-1 (OMM-1) atlas space (Arthofer et al., 2024). Specifically, we proceeded as follows:

- For histology and histology-based dMRI simulations, we first registered two-dimensional tissue masks to the mid-sagittal slice of the FA atlas in OMM-1 space (see Fig. 2a, c, f). Then, we transformed histological ROI centroids to OMM-1 space, assigned histological/simulated $r_{\text{eff}}$ values, and generated continuous spatial patterns using nearest-neighbor interpolation.
- Since each ex-vivo dMRI ROI covered multiple voxels, we first averaged $r_{\text{eff}}$ values within each ROI in native space. We then mapped these averages to OMM-1 space using the same approach as for histology, given that ROIs between these modalities were registered per our study design (see Fig. 2e).
- For in-vivo dMRI, we used both $T_{1}$-weighted image and FA map for registration to corresponding templates in OMM-1 space, using ANTs diffeomorphic registration Avants et al., 2008 with a weighted multi-modal loss combining $T_{1}$ and FA terms (see Fig. 2c, d, g). Using this registration, we transformed per-subject $r_{\text{eff}}$ maps to OMM-1 space. To select voxels inside the corpus callosum, we applied a coarse mask derived from the JHU ICBM-DTI-81 white matter atlas (Mori et al., 2008; Smith et al., 2004) along with an FA threshold (FA  ≥0.65 ). Additionally, we considered only voxels with biologically feasible $r_{\text{eff}}$ values ($r_{\text{eff}}\geq \text{0.1 µm}$ ) (Waxman et al., 1995).

#### 3.6.2 Quantitative comparison of spatial ${\text{r}}_{\text{eff}}$ patterns

To quantitatively compare $r_{\text{eff}}$ from dMRI experiments to histological values, we determined corresponding $r_{\text{eff}}$ values in the respective native spaces.

- For ex-vivo dMRI, we computed the mean $r_{\text{eff}}$ across all voxels per ROI, with ROIs registered to histological ROIs per our study design (see Fig. 2e).
- For in-vivo dMRI, we determined histological ROI coordinates in native dMRI space by first transforming them to OMM-1 space and then mapping them to the nearest voxel in dMRI native space (see Fig. 2a, c, d). For comparison with histological values, we used the group-average $r_{\text{eff}}$ , computed from spatially smoothed per-subject $r_{\text{eff}}$ maps (FWHM = 3.75 mm ), ensuring that spatial smoothing was restricted to corpus callosum voxels (J. E. Lee et al., 2009) (see Supplementary Section S1 for an analysis of smoothing impact). To ensure robust group-averages, we applied the same corpus callosum voxel selection criteria to per-subject $r_{\text{eff}}$ maps as in the qualitative analysis (FA ≥  0.65 , $r_{\text{eff}}\geq \text{0.1 µm}$ ) before averaging and retained only ROIs with at least 3 of 5 contributing subjects.

**Error metrics** Between histological values ($r_{\text{eff}}$ ) and fitted/estimated values (${\hat{r}}_{\text{eff}}$ ) from dMRI experiments as described in Sections 3.2.4 and 3.3.4, we computed a linear regression slope to assess the scaling behavior of ${\hat{r}}_{\text{eff}}$ . Additionally, we determined the fitting success rate

$$S=\frac{1}{N}\sum_{i=1}^{N}I({\hat{r}}_{\text{eff},i}\geq \text{0.1 µm}),$$ (6)

as the proportion of biologically feasible estimates (${\hat{r}}_{\text{eff}}\geq \text{0.1 µm}$ , Waxman et al. (1995)), where N=35 is the number of ROIs and I(⋅) is the indicator function, which equals 1 if the condition inside is true and 0 otherwise. ROIs that did not meet voxel-selection criteria (corpus callosum masking or insufficient subject support for group-averages) were also counted as unsuccessful.

To quantify absolute agreement, we computed the normalized root-mean-square error:

$$\text{NRMSE}=\frac{\sqrt{\sum_{i=1}^{N}{\left({\hat{r}}_{\text{eff},i}-r_{\text{eff},i}\right)}^{2}}}{\sum_{i=1}^{N}r_{\text{eff},i}},$$ (7)

using ${\hat{r}}_{\text{eff},i}=\text{0 µm}$ for unsuccessfully fitted values.

To quantify the ability to capture linear relationships, we computed Pearson’s correlation coefficient:

$$R=\frac{\sum_{i=1}^{N}\left({\hat{r}}_{\text{eff},i}-\langle {\hat{r}}_{\text{eff}}\rangle \right)\left(r_{\text{eff},i}-\langle r_{\text{eff}}\rangle \right)}{\sqrt{\sum_{i=1}^{N}{\left({\hat{r}}_{\text{eff},i}-\langle {\hat{r}}_{\text{eff}}\rangle \right)}^{2}}\sqrt{\sum_{i=1}^{N}{\left(r_{\text{eff},i}-\langle r_{\text{eff}}\rangle \right)}^{2}}},$$ (8)

where $\langle r_{\text{eff}}\rangle$ and $\langle {\hat{r}}_{\text{eff}}\rangle$ denote the mean histological and estimated $r_{\text{eff}}$ values across ROIs. To assess statistical significance, we performed a Monte Carlo permutation test under the null hypothesis that ${\hat{r}}_{\text{eff}}$ and $r_{\text{eff}}$ are uncorrelated (R=0 ). We computed the associated p-value as:

$$p=\frac{1}{K}\sum_{i=1}^{K}I\left(\left|{R^{\prime }}_{i}\right|\geq \left|R\;\right|\right),$$ (9)

where $R^{\prime }i$ were computed from shuffled $r_{\text{eff}}$ and fixed ${\hat{r}}_{\text{eff}}$ to approximate the null distribution, using $K=10^{6}$ permutations.

For dMRI simulations with M=1000 repetitions, we pooled over all M×N values to compute the linear regression, S, R and NRMSE . Accordingly, we computed p over M×K iterations with K=1000 so that $M\times K=10^{6}$.

### 3.7 The origins of the model-inherent bias

The assessment in Section 3.6 revealed a model-inherent bias of dMRI-based $r_{\text{eff}}$ . To investigate the origins of this bias, we assessed the influence of signal approximations involved in deriving $r_{\text{eff}}$ (see Appendix A.1). These approximations, applied in sequence, include:

- the Gaussian phase approximation (GPA; see Equation (A5)),
- the wide pulse approximation (WPA; see Equation (A6)),
- the Taylor approximation (see Equation (A9)),
- the exponential approximation (see Equation (A10)).

We evaluated the accuracy of these approximations at the level of the powder-averaged signal $S^{\circ }(b)$ (see Equation (2))—the signal to which $r_{\text{eff}}$ is fitted. While the Taylor approximation and exponential approximation directly model $S^{\circ }(b)$ , the matrix method, the GPA and the WPA model signals per gradient direction $S(b,\vec{g})$ as per Equation (A1). For the latter methods, we first simulated $S(b,\vec{g})$ and then estimated $S^{\circ }(b)$ using a Gaussian maximum likelihood estimator.

### 3.8 Sensitivity constraints beyond the human corpus callosum

To study $r_{\text{eff}}$ mapping in anatomies beyond our human corpus callosum dataset, we extended simulations to mimic other axon populations by scaling the radius distributions from our data. Specifically, we scaled axon radii to extrapolate axon radius distributions of the rat corpus callosum (scaling factor: 0.5 (Veraart et al., 2020)) and the human corticospinal tract (scaling factor: 1.15 (Veraart et al., 2021)). For in-vivo simulations, these scaling factors were applied alongside the tissue shrinkage compensation factor (1.3). For example, we used the scaling factor 1.15⋅1.3≈1.5 for in-vivo corticospinal tract simulations.

### 3.9 Optimal in-vivo dMRI protocols for ${\text{r}}_{\text{eff}}$ mapping

#### 3.9.1 Goals and setup

We optimized in-vivo dMRI protocols for a range of maximum gradient strengths ($g_{\text{max}}=[40,600]\text{ mT/m}$ ), covering the capabilities of existing clinical and research 3T scanners. The optimization aimed to maximize R between histological $r_{\text{eff}}$ and simulated $r_{\text{eff}}$ for protocol candidates, where the simulations were conducted analogously to those for our experimental protocols described. In contrast to these simulations, we assumed Gaussian rather than Rician noise, as Rician noise more strongly obscures correlation (see Supplementary Section S2) but can, in principle, be mitigated through advanced preprocessing techniques (Eichner et al., 2015; Fan et al., 2020; Manzano Patron et al., 2024).

#### 3.9.2 Definition of the optimization problem

To streamline the parameter search, we formulated the optimization problem using the constraints of our experimental in-vivo dMRI protocol. Specifically, we focused on two-shell protocols with fixed diffusion timing parameters (δ, Δ) and fixed minimum b ($b_{\text{min}}={\text{6 ms/µm}}^{2}$) to suppress extra-axonal signal, while allowing the minimum g ($g_{\text{min}}$ ) to vary. Thus, we modeled the optimization problem as

$$\theta^{*}{|}_{g_{\text{max}}}={\text{argmax}}_{\theta }R(\theta ){|}_{g_{\text{max}}},\;\theta =\left\{\delta ,\Delta ,g_{\text{min}}\right\}.$$ (10)

The search grid for θ and additional parameters are detailed in Table 3. Additionally, we enforced Δ ≥δ+4 ms .

**Table 3.** In-vivo and ex-vivo simulation parameters.

			Ex-vivo simulations			In-vivo simulations			
Parameter	Symbol	Unit	Default value	Comparison across modalities (experiment-like)	Comparison across modalities (idealized)	Default value	Comparison of $r_{\text{eff}}$ across modalities (experiment-like)	Comparison of $r_{\text{eff}}$ across modalities (idealized)	Protocol optimization
signal-to-noise ratio	SNR	-	-	${\left[17,51\right]}^{1}$	$\infty^{2}$	-	$32^{1}$	$\infty^{2}$	Equation (13)
noise distribution	-	-	-	Rician	-	-	Rician	-	Gaussian
powder-average estimator	-	-	-	Rician ML	Gaussian ML	-	Rician ML	Gaussian ML	Gaussian ML
radius scaling factor	-	-	1.0	*	*	$1.3^{3}$	*	*	*
neurite dispersion	-	-	$8.2^{1}$	*	*	$8.2^{1}$	*	*	*
diffusion shells	-	-	$9^{4}$	*	*	$2^{4}$	*	*	*
gradient directions per shell	-	-	$65^{4}$	*	*	${\left\{60,120\right\}}^{4}$	*	*	*
axoplasmic diffusivity	$D_{\text{0}}$	µm^2^/ms	$0.35^{5}$	*	*	$2.07^{6}$	*	*	*
parallel intra-axonal diffusivity	$D_{\text{a}}^{∥}$	µm^2^/ms	$0.35^{5}$	*	*	$2.07^{6}$	*	*	*
minimum b	$b_{\text{min}}$	$\text{ms}/{\text{µm}}^{2}$	$20^{4}$	*	*	$6^{4}$	*	*	*
maximum b	$b_{\text{max}}$	$\text{ms}/{\text{µm}}^{2}$	$100^{4}$	*	*	$30.45^{4}$	*	*	$f{\left(\delta ,\Delta ,g_{\text{max}}\right)}^{7}$
minimum g	$g_{\text{min}}$	mT​/​m	$200^{4}$	*	*	$124^{4}$	*	*	[40,600]
maximum g	$g_{\text{max}}$	mT​/​m	$1278^{4}$	*	*	$279^{4}$	*	*	$f{\left(\delta ,\Delta ,b_{\text{min}}\right)}^{7}$
diffusion gradient time	δ	ms	$7^{4}$	*	*	$15^{4}$	*	*	[2,60]
diffusion gradient separation	Δ	ms	$20.1^{4}$	*	*	$29.25^{4}$	*	*	[6,80]
intra-axonal water fraction	$f_{0}$		$0.41^{6}$	*	*	$0.41^{6}$	*	*	*
immobile water fraction	$f_{\text{im}}$	-	$0.27^{1}$	*	*	$0^{8}$	*	*	*
$T_{2}$-weighted intra-axonal water fraction	$f_{\text{a}}$	-	$0.58^{9}$	*	*	$0.58^{9}$	*	*	Equation (12)
$T_{2}$-weighted extra-axonal water fraction	$f_{\text{e}}$	-	$0.15^{10}$	*	*	$0.42^{10}$	*	*	$f{\left(f_{\text{a}},f_{\text{im}}\right)}^{10}$
extra-axonal signal	$S_{\text{e}}$	-	0	*	*	0	*	*	*
intra-axonal transverse relaxation time	$T_{\text{2,a}}$	ms	$29.4^{11}$	*	*	$82^{6}$	*	*	*
extra-axonal transverse relaxation time	$T_{\text{2,e}}$	ms	$15.8^{11}$	*	*	$44^{6}$	*	*	*
echo time	$T_{\text{E}}$	ms	$34.7^{4}$	*	*	$66^{4}$	*	*	Equation (11)

Annotations denote: *the default value was used; ^1^estimated from our experimental data; ^2^we did not add noise; ^3^estimated as the mean of previously reported values (Aboitiz et al., 1992; Tang et al., 1997); ^4^parameters of our experimental protocols; ^5^reported by West et al. (West et al., 2018); ^6^reported by Veraart et al. (Veraart et al., 2018); ^7^computed from the relation $b=g^{2}\gamma^{2}\delta^{2}(\Delta -\delta /3)$ (Stejskal, 1965; Tanner, 1979); ^8^reported by Tax et al. (Tax et al., 2020); ^9^computed as; $f_{a}=f_{0}e^{-T_{\text{E}}/T_{2,\text{a}}}/{\left(f_{0}e^{-T_{\text{E}}/T_{2,\text{a}}}+(1-f_{0}-f_{\text{im}})e^{-T_{\text{E}}/T_{2,\text{e}}}+f_{\text{im}}\right)}^{10}$ computed as; fe=1−fa−fim   11 we extrapolated $T_{2,a}$ and $T_{2,e}$ values from a 3T scanner (Veraart et al., 2018) to 9.4T by scaling with a conversion factor $T_{2}(\text{9.4T})/T_{2}(\text{3T})\approx \text{30 ms}/\text{83.8 ms}\approx 0.358$ using literature values (Birkl et al., 2014; Murali-Manohar et al., 2020).

#### 3.9.3 Modeling echo time, intra-axonal signal fraction, and SNR

We modeled the effect of the protocol-dependent $T_{\text{E}}$ on $T_{2}$-weighted intra-axonal water fraction ($f_{\text{a}}$) and SNR. The echo time of a protocol candidates was estimated as

$$T_{\text{E}}(\theta )=\delta +\Delta +\text{C},$$ (11)

where the constant C=21 ms , derived from our experimental protocol, accounts for additional contributions, such as the RF pulse and readout gradients. Assuming $f_{\text{im}}=0$ (Tax et al., 2020), we computed $f_{a}$ as

$$f_{\text{a}}(T_{\text{E}})=\frac{f_{0}\cdot e^{-T_{\text{E}}/T_{2,\text{a}}}}{f_{0}\cdot e^{-T_{\text{E}}/T_{2,\text{a}}}+(1-f_{0})\cdot e^{-T_{\text{E}}/T_{2,\text{e}}}},$$ (12)

where $f_{0}=0.41$ is the non-$T_{2}$-weighted intra-axonal water fraction, $T_{2,\text{a}}=\text{82}\;\;\text{ms}$ is the intra-axonal $T_{2}$-value, and $T_{2,\text{e}}=\text{44 ms}$ is the extra-axonal $T_{2}$-value, as reported by Veraart et al. (2018). Using ${\text{SNR}}_{\text{ref}}=32$ and $T_{\text{E},\text{ref}}=\text{66 ms}$ , derived from our experimental protocol as reference values, we extrapolated

$$\text{SNR}(T_{\text{E}})={\text{SNR}}_{\text{ref}}\cdot \frac{f_{0}\cdot e^{-T_{\text{E}}/T_{2,\text{a}}}+(1-f_{0})\cdot e^{-T_{\text{E}}/T_{2,\text{e}}}}{f_{0}\cdot e^{-T_{\text{E},\text{ref}}/T_{2,\text{a}}}+(1-f_{0})\cdot e^{-T_{\text{E},\text{ref}}/T_{2,\text{e}}}}.$$ (13)

To evaluate a potential elevation of the baseline SNR level through technical or acquisition improvements, we repeated the protocol optimization analyses for ${\text{SNR}}_{\text{ref}}$ increased by 75% and 150% , yielding SNR values of 56 and 80 for our experimental protocol.

## 4 Results

### 4.1 An in-vivo dMRI-scale histological reference for $r_{\text{eff}}$

**Fig. 3.**
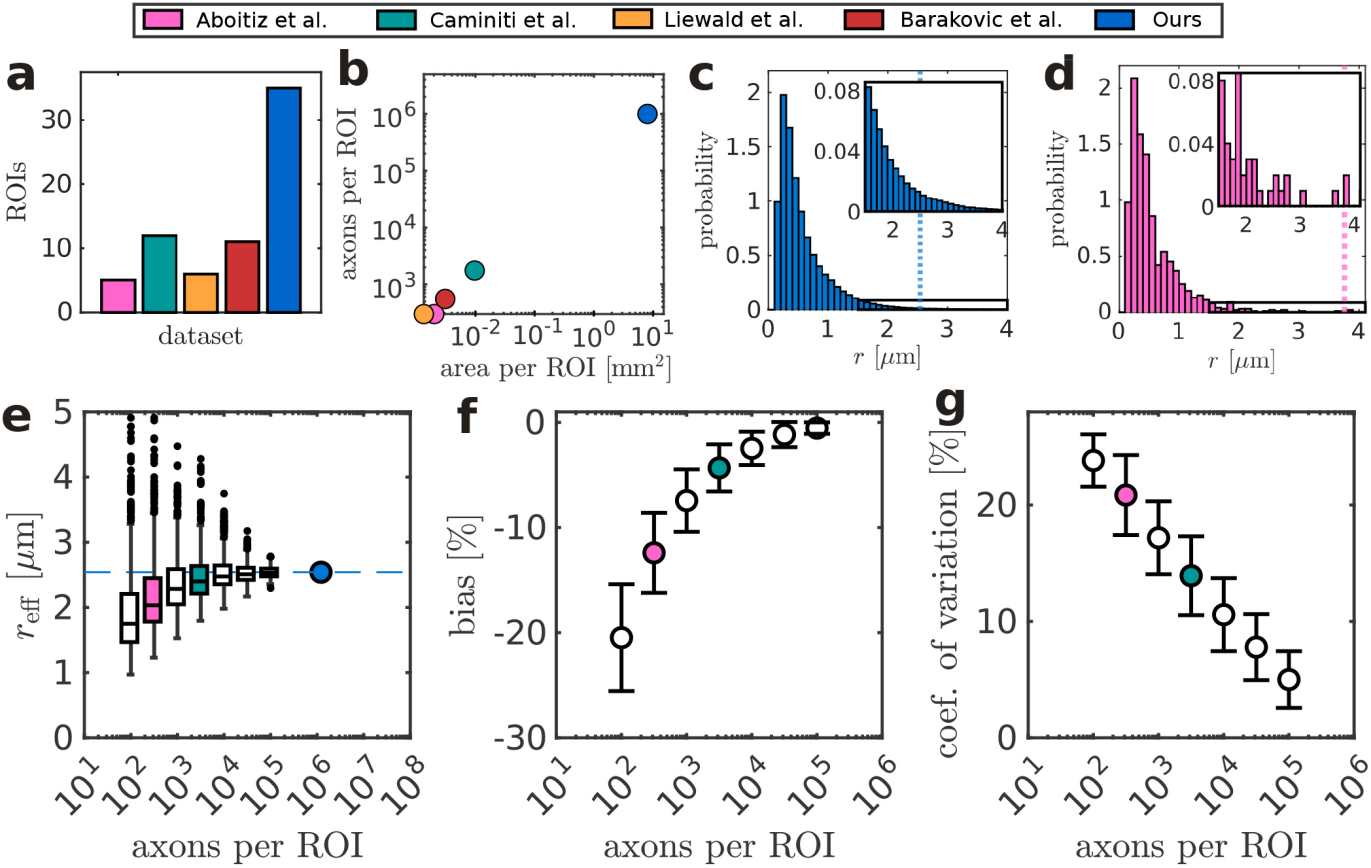
A dMRI-scale histological reference for $r_{eff}$ . (a, b) Sampling statistics of human corpus callosum histology datasets (Aboitiz et al., 1992; Barakovic et al., 2023; Caminiti et al., 2009; Liewald et al., 2014): (a) number of ROIs and (b) mean sample area and axon count per ROI (double-logarithmic scale; area for Barakovic et al. (2023) estimated via linear fit of reported axon counts versus area from Aboitiz et al. (1992), Caminiti et al. (2009), and Liewald et al. (2014)). For Barakovic et al. (2023), we refer to the “Histology2” dataset, which is based on a sample presented in Caminiti et al. (2009). See also Figure 1 for an illustration of ROI sizes. (c, d) Axon radius distribution for (c) a light microscopy ROI and (d) a random subsample of the distribution in (c) including $10^{3}$ axons, mimicking a ROI as presented by Aboitiz et al. (1992). Vertical dotted lines denote $r_{\text{eff}}$ ; insets highlight tails of axon radius distributions. (e) Sampling distribution of $r_{\text{eff}}$ as a function of ROI size (axon count) for the ROI in (c). The blue marker and dashed line represent $r_{\text{eff}}$ computed from all axons within the ROI, while boxplots show simulated sampling distributions for smaller ROI sizes, indicating the median (line), interquartile range (IQR, box), whiskers (1.5 IQR), and outliers (dots). Box colors reflect typical datasets (Aboitiz et al., 1992; Caminiti et al., 2009), categorized by ROI size (see legend). (f, g) Bias and coefficient of variation as a function of the ROI size based on sampling distributions as shown in (e). Markers showing mean ± standard deviation across ROIs. Color encoding follows definitions in (e).

We analyzed 35 light microscopy ROIs from two human corpus callosum samples to establish an in-vivo dMRI-scale histological reference for $r_{\text{eff}}$ . Figure 3 summarizes how our dataset improves spatial sampling, as well as the precision and accuracy of $r_{\text{eff}}$ estimation compared to existing data.

#### 4.1.1 Denser histological sampling across ROIs

Figure 3a and b presents a quantitative comparison of our dataset with existing 2D histology of the human corpus callosum. Our dataset improves spatial sampling both by including a greater number of ROIs and by increasing ROI size, translating into three orders of magnitude more axons per ROI.

#### 4.1.2 Larger ROIs improve accuracy and precision of ${\text{r}}_{\text{eff}}$ through enhanced tail sampling

Figure 3c and d illustrates that light microscopy ROI sizes enable smoother sampling of the tail of the axon radius distribution than ROI sizes used by Aboitiz et al. (1992), which would result in occasional spikes in the tail and deviations in $r_{\text{eff}}$ . This effect of ROI size on $r_{\text{eff}}$ is further explored in Figure 3e, which shows sampling distributions of $r_{\text{eff}}$ computed from repeated sampling at different ROI sizes. Smaller ROIs underestimate $r_{\text{eff}}$ but increase the likelihood of overestimated outliers (see example in Fig. 3d), indicating lower accuracy and precision. The low accuracy and precision of smaller ROIs is quantified across all ROIs in Figure 3f and g. As ROI size increases, both accuracy (bias) and precision (coefficient of variation) improve, with accuracy improving more rapidly. For ROI sizes of existing histology data (Aboitiz et al., 1992; Barakovic et al., 2023; Caminiti et al., 2009; Liewald et al., 2014), the expected bias would be 4 to 12% , whereas the expected coefficient of variation would be 14 to 21% .

### 4.2 Comparison of ${\text{r}}_{\text{eff}}$ across modalities

**Fig. 4.**
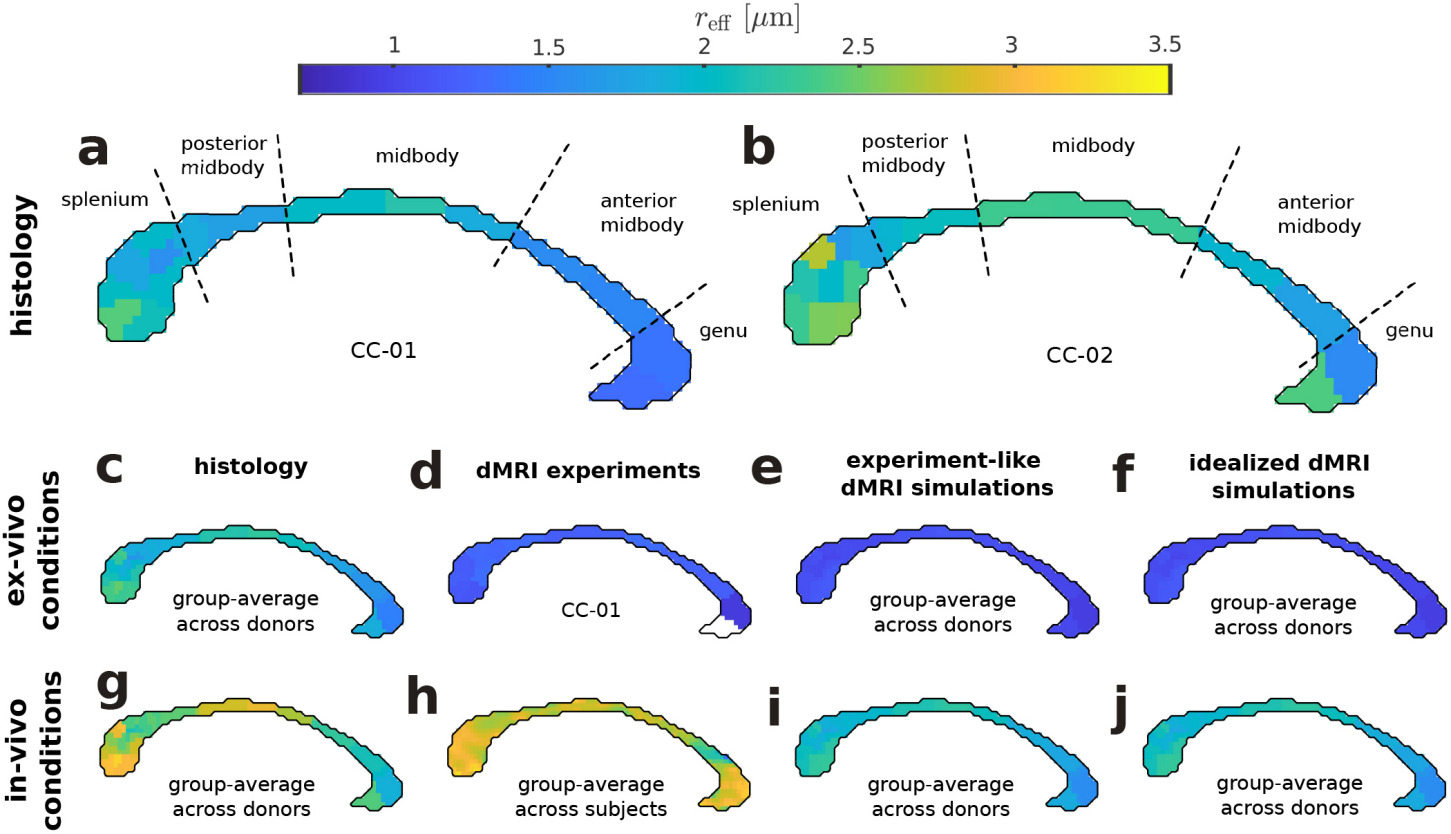
Qualitative comparison of $r_{eff}$ across modalities. (a, b) Histological spatial patterns of $r_{\text{eff}}$ across the corpus callosum, shown in mid-sagittal Oxford-MultiModal-1 (OMM-1, Arthofer et al., 2024) atlas slice with subregions annotated. (c–f) Ex-vivo spatial pattern comparison: (c) histology, (d) dMRI experiments, (e) experiment-like dMRI simulations (experimental SNR), and (f) idealized dMRI simulations (SNR = ∞). Patterns in (c, e, f) show the group-average, whereas (d) covers 15 ROIs of CC-01 (void area not scanned with ex-vivo dMRI; see Fig. 2b, e). For experiment-like simulations in (e), the pattern reflects the median across 1000 repetitions. (g–j) In-vivo spatial pattern comparison analogously to (c–f). Spatial patterns in (g, i, j) are based on histological axon radii scaled by 1.3 to account for tissue shrinkage (Aboitiz et al., 1992; Tang et al., 1997) and pattern in (h) reflects the group-average (see Supplementary Section S5 for per-subject patterns).

**Fig. 5.**
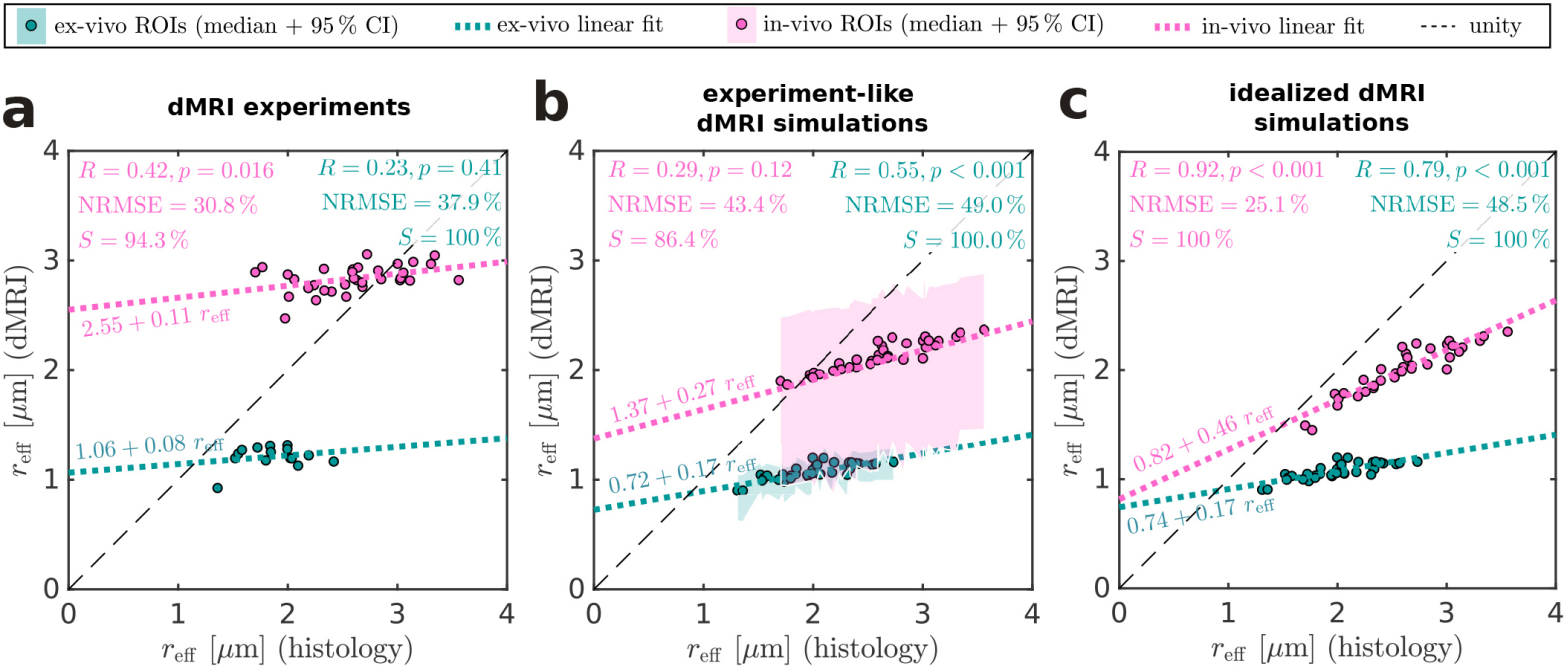
Quantitative comparison of $r_{eff}$ across modalities. (a–c) ROI-wise comparisons of $r_{\text{eff}}$ from dMRI experiments/simulations against histology. Simulations include an (b) experiment-like scenario (experimental SNR) and (c) an idealized scenario (SNR = ∞). Markers represent ROIs in Figure 1a, with colors indicating in-vivo/ex-vivo conditions. For dMRI experiments in (a), ex-vivo markers include 15 ROIs of CC-01 (see Fig. 2b, e), whereas in-vivo markers denote group-average $r_{\text{eff}}$ values (see Supplementary Section S5 for per-subject data). Histological $r_{\text{eff}}$ for in-vivo comparisons in (a–c) are based on axon radii scaled by 1.3 to account for tissue shrinkage (Aboitiz et al., 1992; Tang et al., 1997). Simulations in (b–c) use all histological ROIs and assume a single dMRI subject/donor. 95% confidence intervals (shaded areas in (b)) were computed across 1000 repetitions. Dashed lines illustrate theoretical perfect agreement. Annotated metrics were computed over all ROIs, including Pearson’s correlation coefficient (R) and corresponding p-value, normalized root-mean-square error (NRMSE), and fitting success rate (S) (see Equations (6) to (9)).

To validate $r_{\text{eff}}$ experimentally against our histological reference, we acquired in-vivo and ex-vivo dMRI data. In-vivo, we scanned five healthy human subjects using a high-gradient scanner (300 mT​/​m ), whereas we conducted ex-vivo dMRI scans on tissue sample CC-01 on a preclinical scanner (9.4 T ). As a bridge between histology and dMRI, we conducted dMRI simulations under both idealized (SNR = ∞) and experiment-like conditions with added Rician noise (in-vivo: SNR = 32; ex-vivo: SNR ranging from 17 to 51, depending on the diffusion-weighting b). For a fair comparison between in-vivo dMRI experiments/simulations and histology, we scaled axon radii from histological distributions by 1.3 to account for tissue shrinkage (Aboitiz et al., 1992; Tang et al., 1997). Figure 4 compares spatial $r_{\text{eff}}$ patterns across modalities, whereas Figure 5 provides a quantitative comparison.

#### 4.2.1 Histological samples agree across broader regions but vary locally

Figure 4a and b show the spatial patterns of $r_{\text{eff}}$ in histology across the mid-sagittal section of the corpus callosum. Both samples exhibit similar inter-region trends, with an alternating low-high pattern across the anterior midbody, midbody, posterior midbody, and splenium (see also Supplementary Section S3). However, there is strong intra-region variability within the splenium, inconsistent across tissue samples. In other regions, intra-region variability cannot be conclusively assessed due to sparser sampling.

#### 4.2.2 In-vivo dMRI captures coarse spatial ${\text{r}}_{\text{eff}}$ pattern at reduced dynamic range

Figure 4c–j compare spatial $r_{\text{eff}}$ patterns across the corpus callosum between histology, dMRI experiments and simulations, both for the ex-vivo (see Fig. 4c–f) and in-vivo scenario (see Fig. 4g–j). Ex-vivo dMRI-based $r_{\text{eff}}$ underestimate histological values (see Fig. 4d), aligning with simulations (see Fig. 4e, f). While in-vivo simulations also predict an underestimation of $r_{\text{eff}}$ (see Fig. 4i, j), experimental $r_{\text{eff}}$ overestimate histological values (see Fig. 4h), indicating effects not captured by simulations. Overall, both ex-vivo and in-vivo $r_{\text{eff}}$ patterns exhibit a reduced dynamic range compared to histology, suggesting low sensitivity to microstructure (see Fig. 4d–f, h–j). This low sensitivity complicates the capture of spatial patterns ex-vivo (see Fig. 4d–f), whereas the group-average pattern of in-vivo dMRI experiments (see Fig. 4h) shows some resemblance to histology, hinting at a similar alternating low-high pattern across anterior midbody, midbody, posterior midbody, and splenium. However, the high values in the genu, relative to other regions, do not align with histological patterns. Additionally, partial volume effects may influence the in-vivo dMRI pattern, as suggested by extreme values near border regions, an effect that is likely exacerbated by the relatively large voxel size of our in-vivo acquisition.

#### 4.2.3 Significant correlation with histology in-vivo, but not ex-vivo

The resemblance of the group-average spatial $r_{\text{eff}}$ pattern from in-vivo dMRI experiments with histology is reflected in a significant correlation (see Fig. 5a). However, this analysis exhibited some variability due to the non-deterministic nature of our in-vivo dMRI processing (Fig. 5a shows a representative iteration; over 10 iterations we yielded: R=0.414±0.03 , all p<0.05 ; see Supplementary Section S4). The significant correlation of in-vivo dMRI-based $r_{\text{eff}}$ with histological values was not predicted by our simulations (see Fig. 5b), which, however, assume a single subject rather than a group-average. Supplementary Section S5 provides a more comparable scenario to simulations by showing per-subject correlations, revealing no significant correlation with histology for most individual subjects as predicted by simulations. Yet, in terms of absolute values, there is an offset of about 0.5 µm between $r_{\text{eff}}$ from in-vivo dMRI experiments and simulations (see Fig. 5a, b). For ex-vivo dMRI experiments (see Fig. 5a), there is no significant correlation with histology. This is likely due to reduced precision compared to simulations (see Fig. 5b) and the need to estimate an additional parameter, $f_{\text{im}}$ , which can confound $r_{\text{eff}}$ estimation (see Supplementary Sections S6 and S7).

#### 4.2.4 A model-inherent bias reduces the dynamic range

The idealized dMRI simulations (see Fig. 5c) reveal a primary cause of the reduced dynamic range of dMRI-based $r_{\text{eff}}$ : a proportional bias at larger $r_{\text{eff}}$ , which we refer to as “model-inherent bias”. This bias affects absolute agreement, as measured by the normalized root mean square error (NRMSE), by shifting values below the unity line. Additionally, it reduces R by limiting the dynamic range at the upper end of $r_{\text{eff}}$ values, thereby obscuring correlations under noisy conditions (see Fig. 5a, b). Notably, the model-inherent bias is stronger ex-vivo than in-vivo (see slopes in Fig. 5c).

#### 4.2.5 Low sensitivity to small ${\text{r}}_{\text{eff}}$ additionally reduces the dynamic range

In-vivo experiment-like simulations (see Fig. 5b) show a mild, noise-induced overestimation of smaller $r_{\text{eff}}$ values, affecting sensitivity at the lower end of $r_{\text{eff}}$ values. This reduced sensitivity to small $r_{\text{eff}}$ hints at the practical resolution limit, below which $r_{\text{eff}}$ values may no longer be reliably distinguished from noise (Nilsson et al., 2017).

### 4.3 The origins of the model-inherent bias

**Fig. 6.**
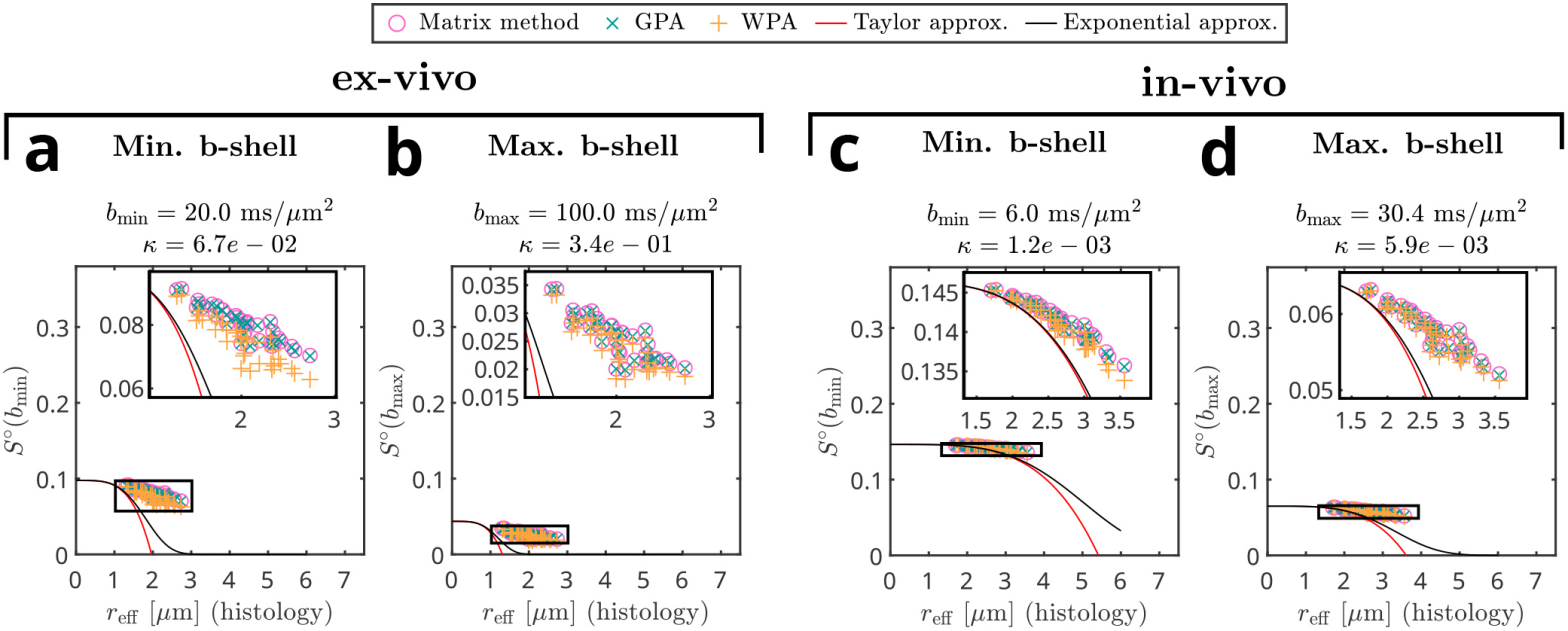
The origins of the model-inherent bias. (a, b) Ex-vivo simulated powder-averaged signals $S^{\circ }(b)$ for: (a) the minimum $b=b_{\text{min}}$ -shell and (b) the maximum $b=b_{\text{max}}$ -shell. Marker symbols/lines represent different $S^{\circ }(b)$ approximations. The matrix method provides the most accurate approach, serving as a reference. All other methods introduce successive simplifications, as arranged from left to right in the legend, ultimately leading to the signal model used for $r_{\text{eff}}$ estimation. Distinct markers correspond to simulations based on axon radius distribution-weighted signals for ROIs (see Fig. 1a). In contrast, lines depict $S^{\circ }(b)$ at later approximation stages, where the axon radius distribution is incorporated into $r_{\text{eff}}$ , allowing it to vary continuously. (c, d) In-vivo simulated powder-averaged signals $S^{\circ }(b)$ , following the same definitions as in (a, b).

The simulations in Figure 5c reveal a model-inherent bias of dMRI-based $r_{\text{eff}}$ . Here, we investigate the origins of this bias by assessing the signal approximations involved in deriving $r_{\text{eff}}$ (see Equations (A5), (A6), (A9), and (A10)). Figure 6 shows the powder-averaged signal $S^{\circ }(b)$ simulated for both in-vivo and ex-vivo experimental MRI protocols, comparing different levels of signal approximation. The matrix method (Callaghan, 1997) is the reference method for our simulations, whereas remaining methods introduce successive simplifications, as arranged from left to right in the legend, ultimately leading to the signal model used for $r_{\text{eff}}$ fitting.

#### 4.3.1 The Taylor approximation drives the model-inherent bias

Both in-vivo and ex-vivo, the Taylor approximation introduces by far the largest deviations between successive approximations, driving the model-inherent bias. The wide-pulse approximation (WPA) also introduces slight deviations, whereas the Gaussian phase approximation (GPA) aligns almost perfectly with our reference method. While relative differences between approximations are preserved across experimental conditions, deviations are generally stronger for the ex-vivo protocol.

#### 4.3.2 The dependency of the model-inherent bias on ${\text{r}}_{\text{eff}}$ and ${\text{g}}_{\text{max}}$

The observed scaling of deviations with $r_{\text{eff}}$ and $g_{\text{max}}$ for the Taylor approximation and the WPA can be understood by examining their underlying dependencies. For the Taylor approximation, accuracy improves when the necessary but not sufficient condition $\kappa r_{\text{eff}}^{4}\ll 1$ is satisfied. While the violation of this condition with increasing $r_{\text{eff}}$ is evident, its violation with increasing g is implicit in the quadratic dependency $g^{2}∼\kappa =\frac{7}{48}\frac{g^{2}\gamma^{2}\delta }{D_{0}}$. Similarly, the deviations caused by the WPA ($\delta >>r^{2}/D_{0}$) are related to both $r_{\text{eff}}$ and $g_{\text{max}}$ . Higher $r_{\text{eff}}$ are linked to the prevalence of larger r, making the assumption less valid, while higher $g_{\text{max}}$ typically allow for shorter δ, further undermining the assumption. In ex-vivo dMRI, the reduced $D_{0}$ further amplifies the deviations due to both Taylor approximation and WPA.

#### 4.3.3 Powder-averaged signals decay near-linearly at high b

Interestingly, $S^{\circ }(b)$ appears to decay almost linearly with $r_{\text{eff}}$ at high b. This behavior suggests an intrinsic property of the signal model, warranting further exploration.

### 4.4 Sensitivity constraints beyond the human corpus callosum

**Fig. 7.**
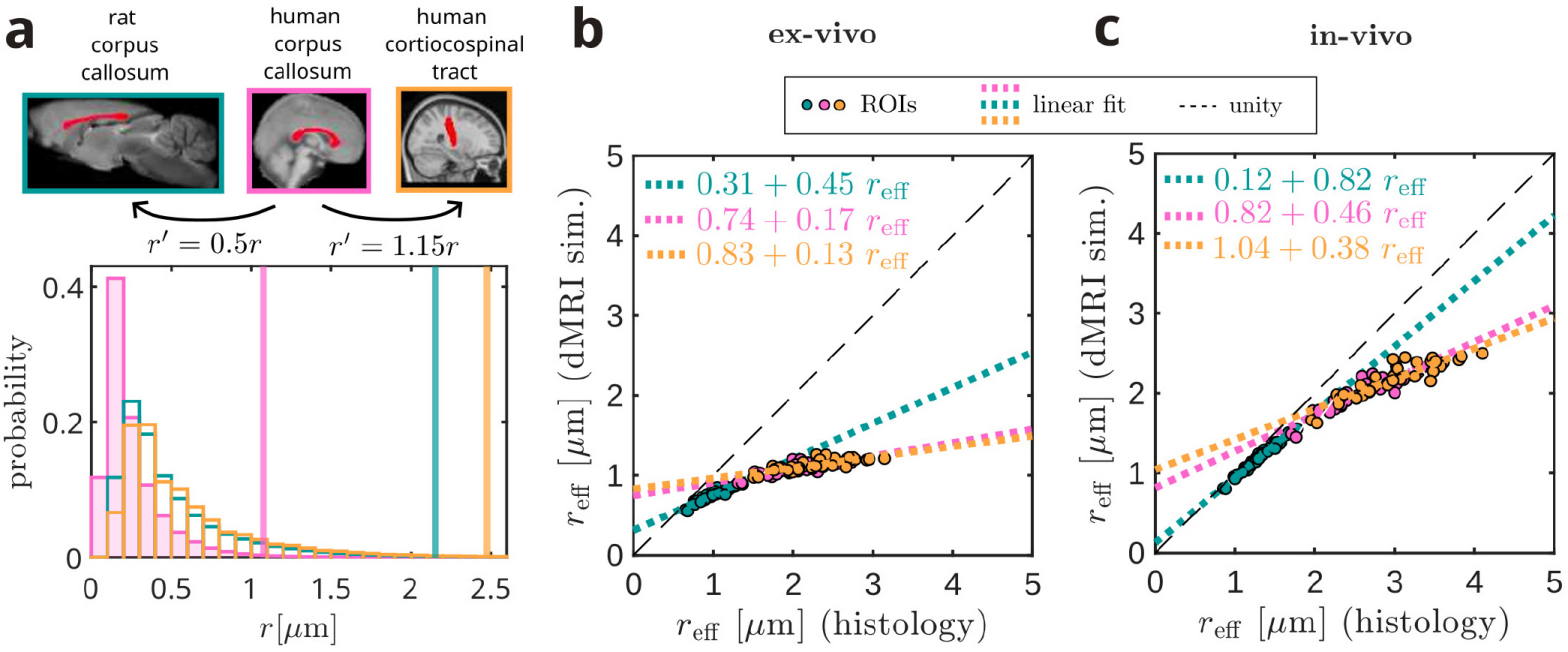
Sensitivity constraints beyond the human corpus callosum. (a) Axon radius distributions for different populations: rat corpus callosum (green), human corpus callosum (magenta), and human corticospinal tract (orange). Vertical lines indicate the corresponding $r_{\text{eff}}$ values. To synthesize axon radius distributions for the rat corpus callosum and human corticospinal tract, we applied literature-derived scaling factors to axon radii from our primary dataset of the human corpus callosum (see annotations). The displayed distributions correspond to the ex-vivo scenario; for in-vivo simulations, an additional scaling factor of 1.3 was applied to account for tissue shrinkage (Aboitiz et al., 1992; Tang et al., 1997). (b, c) ROI-wise comparisons of $r_{\text{eff}}$ from idealized MRI simulations (SNR = ∞) against histological $r_{\text{eff}}$ for each population in (a), evaluated under: (b) the experimental ex-vivo protocol and (c) the experimental in-vivo protocol. Marker/line colors indicate axon population as in (a). Each marker corresponds to a region of interest (ROI) from Figure 1a. Dashed lines denote theoretical perfect agreement, while dotted lines represent linear regressions per axon population.

To investigate how model-inherent bias may affect $r_{\text{eff}}$ measurements outside the human corpus callosum, we extrapolated axon radius distributions from our human corpus callosum dataset to the human corticospinal tract and the rat corpus callosum. We repeated idealized simulations (SNR = ∞) as in Figure 5c, applying additional scaling factors on top of tissue shrinkage correction (only in-vivo; scaling factor: 1.3) to adjust for the target anatomies (rat corpus callosum: 0.5 (Veraart et al., 2020)); human corticospinal tract: 1.15 (Veraart et al., 2021)). Figure 7a shows the resulting axon radius distributions and corresponding $r_{\text{eff}}$ values. Figure 7b and c presents ROI-wise comparisons between simulated and histological $r_{\text{eff}}$ values for these populations, evaluated under both our ex-vivo and in-vivo protocols.

#### 4.4.1 Model-inherent bias scales nonlinearly

Across both in-vivo and ex-vivo conditions (see Fig. 7b, c), the broader $r_{\text{eff}}$ range observed across axon populations reveals a nonlinear scaling of the model-inherent bias. The trend indicates a tendency toward saturation at higher $r_{\text{eff}}$ values—indicating not just a fixed bias, but a loss of sensitivity as $r_{\text{eff}}$ increases.

#### 4.4.2 Sensitivity reduction imposes practical constraints

The trends observed previously—stronger sensitivity reduction under ex-vivo conditions—persist across axon populations. In-vivo (Fig. 7c), this implies that brain regions with larger axons, such as the corticospinal tract, may be particularly affected, potentially imposing anatomical constraints on $r_{\text{eff}}$ mapping. Ex-vivo (see Fig. 7b), sufficient sensitivity appears retained for small-axon populations such as the rat corpus callosum, provided lower $r_{\text{eff}}$ values remain distinguishable from noise under experimental conditions (Nilsson et al., 2017). However, for human white matter, sensitivity is minimal, raising concerns about whether the remaining sensitivity can be meaningfully exploited in real-world ex-vivo dMRI acquisitions.

### 4.5 Optimal in-vivo dMRI protocols for ${\text{r}}_{\text{eff}}$ mapping

**Fig. 8.**
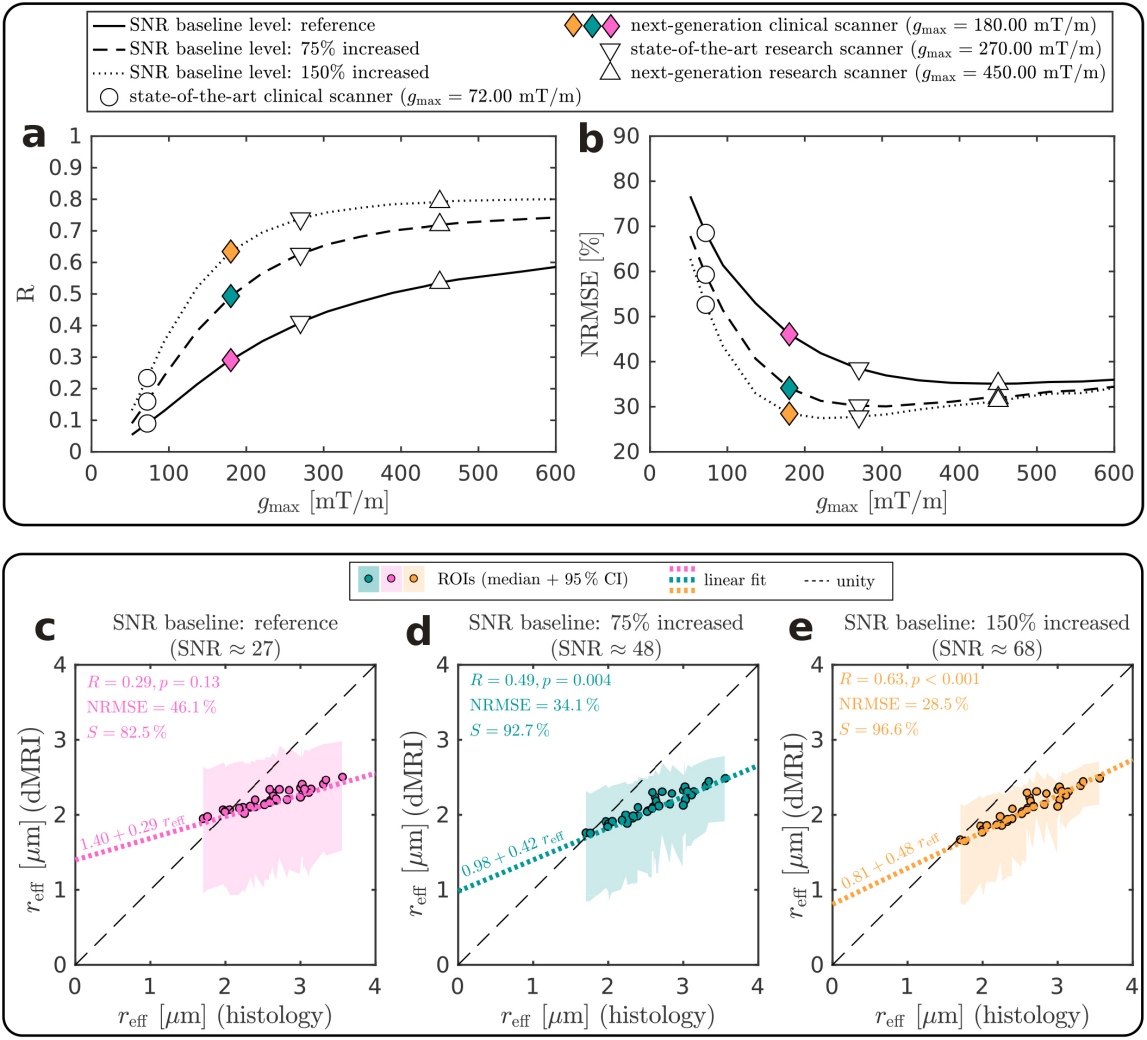
Optimal in-vivo dMRI protocols for $r_{eff}$ mapping. (a, b) Optimal Pearson’s correlation coefficient (R) and normalized root mean square error (NRMSE ) as a function of maximum gradient amplitude ($g_{\text{max}}$ ). Markers encode $g_{\text{max}}$ of existing clinical scanners and research scanners (assuming 90% of the nominal $g_{\text{max}}$ ). Colored markers highlight optimal protocols for next-generation clinical scanners. Line styles indicate different SNR baseline levels. While the reference SNR baseline level reflects our experimental conditions, increased SNR baseline levels assume an SNR increase through potential technical or acquisition advances. In addition, we accounted for SNR variation due to protocol parameter differences (see Equation (13)). For our experimental protocol, baseline SNR levels would correspond to SNR values of 32 (reference), 56 (75% increased), and 80 (150% increased). Note that we optimized protocols by maximizing R, whereas NRMSE is an auxiliary metric. (c–e) Comparison of estimated reff with histological values for optimal next-generation clinical scanner protocols across baseline SNR levels (color coding as in (a, b)). SNR values of protocols are annotated above plots. Markers represent histological ROIs in Figure 1a. The 95% confidence intervals (shaded areas) were computed across 1000 noise realizations. The dashed lines illustrate theoretical perfect agreement. The annotated metrics were computed over all ROIs, including Pearson’s correlation coefficient (R) and the corresponding p-value, the normalized root-mean-square error (NRMSE), and the fitting success rate (S) (see Equations (6) to (9)).

We optimized in-vivo dMRI protocols for next-generation 3T clinical scanners with maximum gradient amplitude ($g_{\text{max}}$ ) up to 200 mT​/​m . To this end, we conducted a grid search for optimal protocol parameters and evaluated protocol candidates by simulating their $r_{\text{eff}}$ estimates for our corpus callosum dataset and maximizing the correlation (R) with histological $r_{\text{eff}}$ , assuming a single dMRI subject. We accounted for SNR variations due to protocol parameter choices, but also considered increased baseline SNR levels, independent of protocol parameters, to explore potential gains achievable through technical or acquisition improvements. In contrast to our dMRI experiments, we assumed Gaussian- rather than Rician-distributed signals, which can be achieved with advanced preprocessing techniques (Eichner et al., 2015; Fan et al., 2019; Manzano Patron et al., 2024). Figure 8 summarizes the protocol optimization results.

#### 4.5.1 Next-generation clinical scanners could narrow gap to research scanners

Figure 8a and b show R and NRMSE as a function of $g_{\text{max}}$ , contextualizing the achievable performance of next-generation clinical scanners in comparison to state-of-the-art 3T clinical scanners, state-of-the-art research scanners as used in our dMRI experiments, and next-generation research scanners, assuming 90% of nominal $g_{\text{max}}$ values. For any baseline SNR level, R converges to a maximum value at a certain minimum $g_{\text{max}}$ , where NRMSE is also optimal or close to optimal. While state-of-the-art clinical scanners consistently perform well below optimal R and NRMSE values, next-generation clinical scanners achieve R values much closer to those of research scanners and can reach near-optimal NRMSE at higher SNR baseline levels. The protocol parameters and further analyses for all optimal protocols referenced in Figure 8a and b are presented in Supplementary Section S8.

#### 4.5.2 Next-generation clinical scanners require SNR gains to reveal correlations

Figure 8c–e show simulated $r_{\text{eff}}$ for optimal next-generation clinical scanner protocol at each baseline SNR level (corresponding to colored markers in Fig. 8a, b). At SNR≈27 , reflecting the expected SNR of the protocol candidate under our experimental conditions, next-generation clinical scanners would not reveal a significant correlation for a single subject with our histology data (R=0.29 , p=0.13 , see Fig. 8c). However, our simulations suggest that a significant correlation could be revealed at SNR≈48 (R=0.49 , $p=3.7e^{-3}$ , see Fig. 8d) and a stronger correlation at SNR≈68 (R=0.63 , p<0.05 , see Fig. 8e).

#### 4.5.3 The model-inherent bias is a relevant factor in protocol design

While R remains stable after reaching the optimum at some $g_{\text{max}}$ (see Fig. 8a), NRMSE increases thereafter (see Fig. 8b). We attribute this loss of absolute agreement to the increasing influence of the model-inherent bias, which drives large $r_{\text{eff}}$ further away from the unity line with increasing $g_{\text{max}}$ (see Supplementary Section S8). Hence, within the employed model’s constraints, model-inherent bias is a relevant factor in protocol design for scanners with very high $g_{\text{max}}$ , such as next-generation research scanners. To fully exploit the potential of these scanners, improved modeling is required.

## 5 Discussion

We addressed the longstanding challenge of quantitatively validating axon radius measurements from diffusion-weighted MRI (dMRI)—a challenge made increasingly pressing by recent advances in acquisition hardware and signal modeling. While previous studies hint at qualitative patterns common with histology, they fall short of quantitatively demonstrating that experimental dMRI captures spatial variation in axon radii—relying instead on simulations, sparsely sampled histology, or histological datasets with insufficient variation to reveal spatial trends. Here, we assessed quantitative spatial correlations between the dMRI-visible effective axon radius ($r_{\text{eff}}$ ) and densely sampled histology from two human corpora callosa. While ex-vivo dMRI showed no significant correlation with histological values, a significant group-level correlation in-vivo provides the first quantitative evidence that dMRI-based axon radius estimates reflect underlying tissue microstructure in the human brain. While we demonstrate this correlation with in-vivo dMRI data acquired on an advanced research scanner, histology-grounded simulations suggest that emerging high-gradient clinical scanners—now available—may bring in-vivo $r_{\text{eff}}$ mapping within reach of clinical adoption, although substantial improvements in SNR remain necessary. In particular, clinical adoption may be facilitated by addressing a newly identified sensitivity limitation in current models—caused by how axon populations are collapsed into a single scalar metric—through future modeling advances.

### 5.1 In-vivo sensitivity persists despite confounds and modeling limitations

From a mechanistic perspective, our findings experimentally demonstrate that a specific axon morphology marker, $r_{\text{eff}}$ , leaves a detectable signature in the in-vivo dMRI signal in the human brain. This sensitivity to $r_{\text{eff}}$ survives despite unmodeled competing intra-axonal signal contributions. In particular, recent 3D histology (M. Andersson et al., 2020; H.-H. Lee et al., 2019; Shapson-Coe et al., 2024; Tian et al., 2025) has challenged the core assumption of perfectly cylindrical axons underlying $r_{\text{eff}}$ , suggesting substantial impact of complex axonal morphology on the dMRI signal in Monte Carlo simulations (M. Andersson et al., 2020, 2022; H.-H. Lee, Papaioannou, et al., 2020; H.-H. Lee et al., 2019, 2024; Winther et al., 2024). In addition, our study reveals another fundamental limitation: a model-inherent proportional bias that reduces sensitivity to $r_{\text{eff}}$ , potentially challenging application in brain regions with very large axons, such as the corticospinal tract (see Figs. 5c and 7). This bias arises from the reduction of the full axon radius distribution to a single scalar, $r_{\text{eff}}$ , and scales with its magnitude (see Fig. 6). As such, we expect it to persist even if $r_{\text{eff}}$ were not only computed from radius distributions across axons, as done here, but also incorporated along-axon radius variations, as recently suggested (H.-H. Lee, Jespersen, et al., 2020).

The sensitivity of in-vivo dMRI to $r_{\text{eff}}$ persists not only in the face of intra-axonal modeling limitations, but also against a broader array of confounding signal contributions, including unmodeled compartment signals (Alexander et al., 2010; Palombo et al., 2020; Pizzolato et al., 2023; Stanisz et al., 1997; Veraart et al., 2019, 2020), relaxation effects (Barakovic et al., 2023), orientation dispersion (M. Andersson et al., 2022; Drobnjak et al., 2016; Nilsson et al., 2012), partial volume effects (Alexander et al., 2001; Vos et al., 2011), and Rician noise bias (Gudbjartsson & Patz, 1995). In addition, in-vivo dMRI-histology comparisons are further affected by tissue deformation and shrinkage (Aboitiz et al., 1992; Dyrby et al., 2018; Tang et al., 1997; Yendiki et al., 2022), inter-individual differences, inter-cohort differences, and scan-rescan variability, although the latter has been reported to be low for the in-vivo protocol we adopted (Veldmann et al., 2024; Veraart et al., 2021).

This myriad of confounding factors suggests that achieving specificity to $r_{\text{eff}}$ is challenging and indeed, our in-vivo data hints at such effects. While there appears to be only a slight overestimation of $r_{\text{eff}}$ compared to histological values, our simulations indicate that the model-inherent bias alone should introduce substantial underestimation. This suggests the presence of an additional overestimation effect of similar magnitude that counteracts the model-inherent bias—highlighting the potential for ambiguities in absolute value range comparisons, such as those conducted in rats (Veraart et al., 2020). The better agreement in absolute values between simulations and ex-vivo dMRI experiments—despite a lack of correlation—raises the possibility that Rician noise bias, more pronounced at the lower SNR of in-vivo dMRI, may drive part of the observed shift. Yet, in light of the complex interplay of additive and compensating confounding effects, attributing such discrepancies to specific sources remains difficult. Hence, given the previously unproven sensitivity of dMRI to $r_{\text{eff}}$ , and more broadly to axonal morphology in the human brain, the next logical step was to provide quantitative experimental evidence for sensitivity.

### 5.2 In-vivo correlation is driven by coarse anatomical pattern

The spatial variation we validate largely reflects a coarse low-to-high pattern across the corpus callosum—spanning the anterior midbody, midbody, posterior midbody, and splenium—as suggested by visual inspection. This pattern aligns most closely with previous findings in humans (Horowitz, Barazany, Tavor, Bernstein, et al., 2015) and nonhuman primates (Caminiti et al., 2009; Lamantia & Rakic, 1990). Differences to regional trends reported in other human studies (Aboitiz et al., 1992; Barakovic et al., 2021, 2023; Caminiti et al., 2009; Huang et al., 2015; Pizzolato et al., 2023; Veraart et al., 2021) may stem from variations in anatomical definitions, ROI placement, acquisition protocols, and analysis methods, but could also partially reflect inter-individual variability in axon morphology. Indeed, the described pattern is visible only at the group-level, whereas individual subjects and donors show notable variability (see Fig. 4a, b for inter-subject differences and Supplementary Section S5 for a quantification of inter-donor differences). A further contributor is the age mismatch between cohorts: in-vivo subjects averaged 31 years, whereas histology donors averaged 61 years. Axon radius is known to increase with age in human white matter (Aboitiz et al., 1996; Fan et al., 2019), but our simulations suggest a small impact on correlations based on an age-related change estimated from a dMRI study (Fan et al., 2019) (see Supplementary Section S9). While part of the variability across subjects and donors may be due to imperfect alignment across subjects and modalities, these findings raise the question of whether finer-grained common spatial patterns exist at all. In light of this variability and the limited number of histology donors in our study, confirming sensitivity of in-vivo dMRI to $r_{\text{eff}}$ on independent datasets remains among the most immediate priorities.

### 5.3 Ex-vivo validation appears challenging

Ex-vivo dMRI is often used as an intermediate step toward in-vivo validation, as it excludes inter-individual differences and allows higher resolution than in-vivo dMRI; however, our results suggest it introduces its own challenges. Unlike in-vivo, we found no correlation ex-vivo. This may be partly due to the smaller number of ROIs available (15 ex-vivo vs. 35 in-vivo), but more fundamentally our simulations show that model-inherent bias strongly reduces sensitivity to $r_{\text{eff}}$ under typical ex-vivo conditions, as it scales with gradient amplitude and decreased $D_{0}$. While ex-vivo dMRI measurements may retain sufficient sensitivity in species with predominantly small axons, such as rats, the sensitivity in human white matter appears prohibitively low. Consequently, validation studies performed in rats (Barazany et al., 2009; Veraart et al., 2020) are limited not only by anatomical differences from humans (Leenen et al., 1982), but also by fundamentally different sensitivity constraints. For the specific protocol we assessed, the sensitivity to $r_{\text{eff}}$ leaves little headroom to detect correlations if additional unaccounted for effects are present. One strong candidate for such an effect is the presence of an immobile water compartment in ex-vivo tissue, which introduces an additional parameter estimation step—along with its associated uncertainty. Our simulations suggest this uncertainty has stronger impact ex-vivo than in-vivo (see Supplementary Sections S6 and S7). Recent methods propose addressing this confound by modeling the spherical variance rather than the spherical mean of the dMRI signal (Pizzolato et al., 2023; Veraart, Raven et al., 2023), an approach that could be evaluated in future studies.

### 5.4 Large-scale 2D histology as a method for experimental ${\text{r}}_{\text{eff}}$ validation

As a secondary outcome, the correspondence of $r_{\text{eff}}$ between in-vivo dMRI and our 2D histology suggests that local axon radius distributions from 2D cross-sections are somewhat representative of the full 3D voxel environment forming the dMRI signal. This interpretation aligns with findings from recent 3D histology (M. Andersson et al., 2020), which indicate that axon radius distributions—and by extension $r_{\text{eff}}$ —may remain relatively stable along fiber bundles, though this has so far only been shown for a limited number (∼50 ) of large axons. Moreover, our approach captures these axon radius distributions more comprehensively than existing 2D histology, as it samples entire cross-sections of in-vivo dMRI voxels rather than small fractions (Aboitiz et al., 1992; Barakovic et al., 2023; Caminiti et al., 2009; Liewald et al., 2014), yielding more accurate (up to 12% ) and precise (up to 21% ) $r_{\text{eff}}$ estimates. These improvements in accuracy and precision are based on subsampling our large-scale axon radius distributions under the assumption of zero spatial autocorrelation. Deviations from this assumption would alter the variance of $r_{\text{eff}}$ estimates, increasing it if large axons cluster and decreasing it if they are evenly dispersed. In conclusion, our 2D histology dataset represents a substantial advance over existing human 2D datasets (Aboitiz et al., 1992; Barakovic et al., 2023; Caminiti et al., 2009; Liewald et al., 2014), providing unprecedented spatial sampling with access to 46 million axons across 35 ROIs. While current 3D histology efforts (M. Andersson et al., 2020; H.-H. Lee et al., 2019; Shapson-Coe et al., 2024; Tian et al., 2025) uniquely enable dMRI simulations (M. Andersson et al., 2020, 2022; H.-H. Lee, Papaioannou, et al., 2020; H.-H. Lee et al., 2019, 2024; Tian et al., 2025; Winther et al., 2024) based on a few hundred complex axonal reconstructions, our large-scale 2D histology approach offers a complementary and scalable route for practical validation of real-world dMRI measurements.

### 5.5 Toward clinical translation with next-generation clinical scanners

Axon radius measurements have so far been restricted to advanced research scanners with gradient amplitudes of up to 300 mT​/​m , as used in our study. In contrast, standard clinical scanners typically operate at gradient strengths up to 80 mT​/​m . However, emerging high-gradient clinical systems—now available on the market—narrow this gap with amplitudes up to 200mT​/​m . In histology-informed protocol optimization simulations, we show that these scanners could approach the performance of research systems for $r_{\text{eff}}$ mapping, provided future advances can improve SNR. Yet, these advances would have to be substantial: to support a significant subject-level correlation (R=0.49 , $p=3.7\times 10^{-3}$ ) with our histology, simulations indicate that a ∼75% SNR increase would be required, reaching SNR≈48 . These requirements may be even higher when unmodeled signal components reduce correlation strength or when Gaussian signal distributions cannot be enforced through advanced preprocessing techniques (Eichner et al., 2015; Fan et al., 2020; Manzano Patron et al., 2024). Overall, these SNR requirements pose a substantial barrier and indicate that near-term clinical translation remains challenging. This is especially true for robust application at the individual-subject level, where our supplementary analyses showed considerable variability in correlations (see Supplementary Section S5). Nonetheless, compared to the prohibitive SNR demands of current clinical systems (Huang et al., 2015; Nilsson et al., 2017; Sepehrband, Alexander, Kurniawan, et al., 2016; Veraart et al., 2020), next-generation clinical scanners with higher gradient amplitudes offer a realistic avenue for translation, provided that further methodological and hardware advances can deliver the necessary SNR gains.

As for candidates to increase SNR, enforcing Gaussian-distributed signals (Eichner et al., 2015; Fan et al., 2020; Manzano Patron et al., 2024) could enable effective SNR gains through denoising, which we did not apply in our experiments or simulations, in order to preserve Rician-distributed signals for Rician maximum likelihood fitting (Varadarajan & Haldar, 2015). Importantly, the identified model-inherent bias highlights a key target for future modeling advances that could help relax SNR demands. Recent developments also promise future SNR gains, for example, a 30% SNR gain was recently demonstrated using advanced acquisition techniques for the same in-vivo protocol applied in our study (Veldmann et al., 2024). Looking further ahead, improvements may arise from ongoing innovations in coil design and gradient hardware (Feinberg et al., 2023; Niendorf et al., 2015). Encouragingly, our simulations in Supplementary Section S10 suggest that meaningful clinical applications could become feasible if such SNR gains are achieved—illustrated by successful discrimination between individuals with autism spectrum disorder and healthy controls within realistic group sizes.

### 5.6 Insights from data-driven protocol design

Expanding beyond clinical protocols, we provide broader insights into the design of in-vivo $r_{\text{eff}}$ measurements, exploring gradient amplitudes ranging from state-of-the-art clinical scanners with 80 mT​/​m to next-generation research systems with 500 mT​/​m . Previous studies have primarily evaluated scanner performance in terms of achievable precision as a function of gradient amplitude and noise, typically assuming idealized models (Huang et al., 2015; Nilsson et al., 2017; Sepehrband, Alexander, Kurniawan, et al., 2016; Veraart et al., 2021). In contrast, our data-driven approach uses histology-informed simulations, capturing both sensitivity reduction and loss of accuracy due to model-inherent bias. This bias increases with both gradient amplitude and $r_{\text{eff}}$ , degrading absolute agreement as measured by normalized root-mean-square error (NRMSE). By contrast, higher gradient amplitudes tend to improve correlation (R) through improved precision, aligning with previous findings (Huang et al., 2015; Nilsson et al., 2017; Sepehrband, Alexander, Kurniawan, et al., 2016; Veraart et al., 2021). However, these gains in R become marginal for gradient amplitudes beyond 300 mT​/​m in regimes of high SNR and large $r_{\text{eff}}$ , at the expense of reduced accuracy. This becomes particularly pronounced for next-generation research scanners with gradient amplitudes of 500 mT​/​m , suggesting that further improvements in modeling are required to fully harness the potential of these scanners.

### 5.7 Limitations and future directions

We focused exclusively on $r_{\text{eff}}$ , a scalar metric with a direct analytic relation to the axon radius distribution, albeit one that is dominated by its large-axon tail. A less tail-weighted scalar metric than $r_{\text{eff}}$ can be defined in the narrow-pulse limit (Burcaw et al., 2015; Sepehrband, Alexander, Kurniawan, et al., 2016), although practical measurements are technically demanding (Nilsson et al., 2017). Other strategies attempt to recover full distributions through parametric fits (Assaf et al., 2008), yet the validity of the assumed distributional forms remains uncertain (Sepehrband, Alexander, Clark, et al., 2016). Even if such assumptions were correct, the additional degrees of freedom in the fit and the disproportionate sensitivity of the dMRI signal to larger axons (Burcaw et al., 2015; Neuman, 1974; Van Gelderen et al., 1994) constrain robust inference about the body of the distribution. With regard to clinical translation, the sensitivity of $r_{\text{eff}}$ to large axons may be advantageous, as large axons are preferentially affected in several neurological disorders (Judson et al., 2017; Stassart et al., 2018; Wegiel et al., 2018; Zikopoulos & Barbas, 2010). Nonetheless, the usefulness of $r_{\text{eff}}$ as a biomarker in pathological tissue remains to be better understood, as alterations may reflect distinct effects such as caliber variations, beadings, or undulations (M. Andersson et al., 2020, 2022; H.-H. Lee, Papaioannou, et al., 2020; H.-H. Lee et al., 2019, 2024).

We applied spatial smoothing to in-vivo dMRI-based $r_{\text{eff}}$ maps to mitigate alignment inaccuracies and reduce apparent noise in comparisons with histology (see Supplementary Section S1). This noise is partly a consequence of our interpolation-efficient pipeline, which applies all spatial transformations in a single resampling step, preserving not only fine-grained spatial detail but also noise. While smoothing reduces sensitivity to subtle spatial variation, substantial inter-individual differences already raise the question of whether such common structure exists at all.

For $r_{\text{eff}}$ estimation, we assume a fixed literature value for the axoplasmic diffusivity ($D_{0}$), which could vary across brain regions and individuals (Veraart et al., 2018). Our simulations in Supplementary Section S11 suggest that realistic variation of $D_{0}$ has only a mild impact on dMRI-histology correlations. Beyond $D_{0}$, we also fixed additional parameters across all voxels in simulations, such as, for example, axonal volume fraction, which cannot capture voxel-wise heterogeneity and may calibrate outcomes to specific tissue regimes.

A further consideration is how individual radii are derived from 2D histology cross-sections. Various approximations exist, most commonly the radius of a circle with equivalent area (used in this study) or the minor axis of a fitted ellipse (Abdollahzadeh et al., 2021; Aboitiz et al., 1992; M. Andersson et al., 2020; Barakovic et al., 2023; Caminiti et al., 2009; Liewald et al., 2014; West et al., 2016). In Supplementary Section S12, we show that $r_{\text{eff}}$ based on these two approximations are closely aligned, and their choice has little impact on experimental validation results. In contrast, ellipse major axes (Veraart et al., 2020) produced inflated $r_{\text{eff}}$ values and no significant correlation with group-level in-vivo dMRI, likely reflecting artifacts from non-orthogonal sectioning rather than true axonal morphology (see example in Mordhorst et al. (2022)).

For protocol optimization, we assumed that Gaussian-distributed signals can be achieved through advanced preprocessing techniques (Eichner et al., 2015; Fan et al., 2020; Manzano Patron et al., 2024). However, such methods are not yet widely adopted in practice, and deviations from the Gaussian assumption could further increase the SNR demands of the proposed next-generation clinical scanner protocols. See Supplementary Section S2 for a comparison of Rician and Gaussian noise in our experimental protocols.

We used relatively large voxels in our in-vivo dMRI acquisition compared to our ex-vivo acquisition. This is a common trade-off to account for the high SNR demands of in-vivo dMRI-based axon radius mapping at strong diffusion-weighting (Fan et al., 2019, 2020; Veldmann et al., 2024; Veraart et al., 2020, 2021).

To account for tissue shrinkage for comparison with in-vivo dMRI, we applied a uniform scaling of axon radius distributions. While this may oversimplify biological reality (Horowitz, Barazany, Tavor, Yovel, et al., 2015) and does not capture potential changes in fiber orientation (Yendiki et al., 2022) or non-linear shrinkage of the extra-axonal space (Dyrby et al., 2018), our correlation analysis inherently accommodates such systematic effects.

Refining the signal model appears to be an immediate avenue for improvement—either by incorporating higher-order terms in Equation (A9) or by exploring the apparent linear decay of $S^{\circ }(b)$ with $r_{\text{eff}}$ (see Fig. 6).

Finally, our open-access histology dataset can serve as a benchmark for studies assessing dMRI-based axon radius estimation beyond the particular metric evaluated ($r_{\text{eff}}$ ). Even beyond the realm of dMRI, it may support efforts to better characterize the tail of the axon radius distribution using parametric descriptions (Sepehrband, Alexander, Clark, et al., 2016).

## 6 Conclusion

We provide the first quantitative evidence that in-vivo MRI-visible axon radius estimates reflect underlying microstructure in the human brain. This quantitative proof of sensitivity to axon morphology marks a critical milestone. At the same time, we outline limitations of our validation design, underscoring the need for independent replication. To support such efforts, we release an open-access histological dataset comprising 46 million axons across 35 ROIs for experimental validation. Our findings further suggest that the newest generation of clinical scanners may support clinical adoption with future technical and modeling advances. Overall, our results motivate continued model development and exploration of potential applications of $r_{\text{eff}}$ as a neuroimaging biomarker.

## Supplementary Material

Supplementary Material

## Data Availability

The processed histology and raw ex-vivo dMRI data are publicly available at https://doi.org/10.5281/zenodo.17431227. The in-vivo dMRI data are available from the corresponding author upon reasonable request. The dMRI processing and simulation code is publicly available.^1^ Our code made use of other publicly available packages, such as MRtrix3 (Tournier et al., 2019), FSL (Smith et al., 2004), the Standard Model Imaging (SMI) toolbox (Coelho et al., 2022, 2023; Novikov et al., 2018; Reisert et al., 2017), the Microstructure Imaging Sequence Simulation ToolBox (MISST) (Drobnjak et al., 2011; Ianuş et al., 2016), and the Advanced Normalization Tools (ANTs) (Avants et al., 2008).

## Appendix

### A.1. Theory

#### A.1.1. Multi-compartment signal model

In white matter, consisting of densely packed, cylindrical axons with radius distribution H(r) , the dMRI signal for one diffusion gradient direction $\vec{g}={\left[g_{\text{x}},g_{\text{y}},g_{\text{z}}\right]}^{T}$ can be written as a volume-weighted average signal (Packer & Rees, 1972)

$$S(b,\vec{g},H(r))=\frac{\int_{r=0}^{\infty }H(r)r^{2}S(b,\vec{g},r)dr}{\int_{r=0}^{\infty }H(r)r^{2}dr},$$
(A1)

where π and the common cylinder length cancel out. When normalized to the signal at b=0 , $S(b,\vec{g},r)$ can be modeled as a multi-compartment signal (Veraart et al., 2019)

$$\begin{array}{l} S(b,\vec{g},r)=\underset{S_{a}(b,\vec{g},r)}{\underset{︸}{f_{a}\;\cdot \;\int_{|\vec{n}|=1}P(\vec{n})\;\underset{S_{a}^{\left|\right|}(b,\xi (\vec{g},\vec{n}),D_{a}^{\left|\right|})}{\underset{︸}{e^{-bD_{a}^{\left|\right|}\xi^{2}}}}\;\underset{S_{a}^{⊥}(b,\xi (\vec{g},\vec{n}),r)}{\underset{︸}{e^{-}bD_{a}^{⊥}(r)(1-\xi^{2})+ο(b^{2})}}d\vec{n}}}\\ \;\;\;\;\;\;\;\;\;\;\;\;\;\;\;\;\;\;\;\;\;\;\;\;\;\;+\underset{S_{e}(b,\vec{g})}{\underset{︸}{f_{e}\cdot \int_{|\vec{n}|=1}P(\vec{n})\underset{S_{e}^{\left|\right|}(b,\xi (\vec{g},\vec{n}),D_{e}^{\left|\right|})}{\underset{︸}{e^{-bD_{e}^{\left|\right|}\xi^{2}}}}\underset{S_{e}^{⊥}(b,\xi (\vec{g},\vec{n}),D_{e}^{⊥})}{\underset{︸}{e^{-}bD_{e}^{⊥}(1-\xi )}}d\vec{n}}}\\ \;\;\;\;\;\;\;\;\;\;\;\;\;\;\;\;\;\;\;\;\;\;\;\;\;\;+f_{im}\prime \end{array}$$
(A2)

with $T_{2}$-weighted intra- and extra-axonal compartment signal fractions ($f_{\text{a}}$, $f_{\text{e}}$) and the signal of immobile water compartment $f_{\text{im}}$ (Stanisz et al., 1997) summing up to 1. $S_{\text{a}}(b,\vec{g},r)$ and $S_{\text{e}}(b,\vec{g})$ are the intra- and extra-axonal compartment signals, which can be modeled as a convolution between the fiber orientation function $P(\vec{n})$ normalized to $\int P(\vec{n})d\vec{n}=1$ and the signal of a fiber pointing in direction $\vec{n}$ (Novikov et al., 2018; Veraart et al., 2019); the latter signal can be represented as the product of parallel ($S_{\text{a}}^{∥}(b,\xi (\vec{g},\vec{n}),D_{\text{a}}^{∥})$ and $S_{\text{e}}^{∥}(b,\xi (\vec{g},\vec{n}),D_{\text{e}}^{∥})$ ) and perpendicular signals ($S_{\text{a}}^{⊥}(b,\xi (\vec{g},\vec{n}),D_{\text{a}}^{⊥}(r))$ and $S_{\text{e}}^{⊥}(b,\xi (\vec{g},\vec{n}))$ ) under the assumption of axisymmetric diffusion, where $\xi (\vec{g},\vec{n})$ denotes the scalar product between $\vec{g}$ and $\vec{n}$ . $S_{\text{a}}^{∥}(b,\xi (\vec{g},\vec{n}),D_{\text{a}}^{∥})$ , $S_{\text{e}}^{∥}(b,\xi (\vec{g},\vec{n}))$ and $S_{\text{e}}^{⊥}(b,\xi (\vec{g},\vec{n}))$ are determined by the scalar diffusivities ($D_{\text{a}}^{∥}$, $D_{\text{e}}^{∥}$ and $D_{\text{e}}^{⊥}$) and the diffusion-weighting $b=g^{2}\gamma^{2}\delta^{2}(\Delta -\delta /3)$ , where γ is the gyromagnetic ratio, g is the diffusion gradient amplitude, δ is the diffusion gradient duration, and the Δ is the diffusion gradient separation (Stejskal, 1965; Tanner, 1979). $S_{\text{a}}^{⊥}(b,\xi (\vec{g},\vec{n}),r)$ can be modeled as a function of r; we introduce several approximations for $S_{\text{a}}^{⊥}(b,\xi (\vec{g},\vec{n}),r)$ in the next section.

#### A.1.2. Perpendicular signal attenuation inside a cylinder

There is no known analytical solution for the $O(b^{2})$ terms in

$$\text{ln}\;\;S_{\text{a}}^{⊥}(b,\xi (\vec{g},\vec{n}),r)=-(1-\xi^{2})bD_{\text{a}}^{⊥}(r)+O(b^{2})$$
(A3)

for finite δ. In the Gaussian phase approximation (GPA), the $O(b^{2})$ terms are neglected and the equation for a cylindrical geometry can be written as (Van Gelderen et al., 1994)

$$\text{ln}\;S_{\text{a,GPA}}^{⊥}(b,\xi (\vec{g},\vec{n}),r)\approx \;-(1-\xi^{2})bD_{\text{a}}^{⊥}(r)$$
(A4)

$$\begin{array}{l} \approx -(1-\xi^{2})\frac{2g^{2}\gamma^{2}r^{4}}{D_{0}}\sum_{m=1}^{\infty }\frac{t_{c}}{\alpha_{m}^{6}(\alpha_{m}^{2}-1)}\\ \cdot \;\;\left[2\alpha_{m}^{2}\frac{\delta }{t_{c}}-2+2e^{-\alpha_{m}^{2}\frac{\delta }{t_{c}}}+2e^{-\alpha_{m}^{2}\frac{\Delta }{t_{c}}}-e^{-2\alpha_{m}^{2}\frac{\Delta -\delta }{t_{c}}}-e^{-2\alpha_{m}^{2}\frac{\Delta +\delta }{t_{c}}}\right], \end{array}$$
(A5)

where the b-dependence is captured implicitly through g, δ, and Δ; $t_{c}=r^{2}/D_{0}$ is the correlation time; $D_{0}$ is the diffusivity of the axoplasm; and $\alpha_{m}$ is the m-th root of $\text{d}J_{1}(\alpha )/\text{d}\alpha =0$ , where $J_{1}(\alpha )$ is the Bessel function of the first kind. In the wide-pulse approximation (WPA), $\Delta >\delta \gg t_{c}$ (Neuman, 1974), the dependency of Equation (A5) on Δ can be neglected so that

$$\text{ln}\;S_{\text{a},\text{WPA}}^{⊥}(b,\xi (\vec{g},\vec{n}),r)\approx \;-(1-\xi^{2})\kappa r^{4},\;\;\kappa =\frac{7}{48}\frac{g^{2}\gamma^{2}\delta }{D_{0}}.$$
(A6)

#### A.1.3. Axon radius estimation via powder-averaged signals

The orientation dependence and interference of $S_{\text{e}}(b,\vec{g})$ in Equation (A2) complicate establishing a robust connection to H(r) . To mitigate interference of $S_{\text{e}}(b,\vec{g})$ , one can study signals in the high b-regime, where its contribution has been reported to be negligible ($b\geq {\text{6 ms/µm}}^{2}$ for in-vivo dMRI (Veraart et al., 2019, 2020) and $b\geq {\text{20ms/µm}}^{2}$ for ex-vivo dMRI (Veraart et al., 2020)). To mitigate orientation dependency, powder-averaging can be applied (Jespersen et al., 2013; Kaden et al., 2016; Mollink et al., 2017). Combining these measures, the powder-averaged signal at sufficiently high b can be approximated as

$$S^{\circ }(b,H(r))\approx \frac{\beta }{\sqrt{b}}\cdot \underset{S_{\text{a}}^{⊥,\circ }\left(b,H\left(r\right)\right)}{\underset{︸}{e^{-bD_{\text{a}}^{⊥}}}}+f_{\text{im}},\;\;\beta =\sqrt{\frac{\pi }{4D_{\text{a}}^{∥}}}f_{\text{a}}.$$
(A7)

The perpendicular component of this signal, $S_{\text{a}}^{⊥,\circ }(b,H(r))$ , can be further simplified through a series of approximations (Burcaw et al., 2015; Veraart et al., 2020)

$$\begin{array}{l} S_{\text{a}}^{⊥,\circ }(b,H(r))\;\;\approx \frac{\int_{r=0}^{\infty }H(r)r^{2}S_{\text{a,WPA}}^{⊥,\circ }(b,r)dr}{\int_{r=0}^{\infty }H(r)r^{2}dr},\;\;S_{\text{a},\text{WPA}}^{⊥,\circ }(b,r)=S_{\text{a},\text{WPA}}^{⊥}(b,\xi =0,r)\;\;\;\;\\ \;\;\;\;\;\;\;\;\;\;\;\;\;\;\;\;\;\;\;\;\;\;\;\;\;\;\;\;\;\;\;=\frac{\int_{r=0}^{\infty }H(r)r^{2}e^{-\kappa r^{4}}dr}{\int_{r=0}^{\infty }H(r)r^{2}dr}\\ \;\;\;\;\;\;\;\;\;\;\;\;\;\;\;\;\;\;\;\;\;\;\;\;\;\;\;\;\;\;\;=\frac{\langle r^{2}(1-\kappa r^{4}+O(r^{8}))\rangle }{\langle r^{2}\rangle } \end{array}$$
(A8)

$$\approx 1-\kappa \frac{\langle r^{6}\rangle }{\langle r^{2}\rangle }$$
(A9)

$$\approx e^{-\kappa r_{\text{eff}}^{4}}$$
(A10)

$$=S_{\text{a},\text{WPA}}^{⊥,\circ }(b,r_{\text{eff}})$$
(A11)

including the WPA in Equation (A8), a Taylor series approximation in Equation (A9), and an exponential approximation in Equation (A10). Here,

$$r_{\text{eff}}=\sqrt[{4}]{\frac{\langle r^{6}\rangle }{\langle r^{2}\rangle }}.$$
(A12)

is a scalar, tail-weighted statistic of H(r) . Alternatively, $r_{\text{eff}}$ can be expressed in terms of dMRI parameters and diffusivities from the relation $e^{-bD_{\text{a}}^{⊥}}\approx e^{-\kappa r_{\text{eff}}^{4}}$ (see Equations (A7) and (A10)), yielding

$$r_{\text{eff}}\approx \sqrt[{4}]{\frac{48}{7}\delta \left(\Delta -\frac{\delta }{3}\right)D_{\text{a}}^{⊥}D_{0}}.$$
(A13)

Using Equations (A7) and (A13), one can jointly estimate $r_{\text{eff}}$ and β, for example, via non-linear fitting (Veraart & Novikov, 2019). If only two different b ($b_{\text{min}}$ and $b_{\text{max}}$ ) are used, one can directly determine

$$D_{a}^{⊥}=\frac{log\left(\frac{S^{\circ }(b_{\text{min}},H(r))}{S^{\circ }(b_{\text{max}},H(r))}\sqrt{\frac{b_{\text{min}}}{b_{\text{max}}}}\right)}{b_{\text{max}}-b_{\text{min}}}$$
(A14)

and subsequently determine $r_{\text{eff}}$ (Pizzolato et al., 2023).

### A.2. dMRI Signal Simulations

We simulated dMRI signals $S(b,\vec{g},H(r))$ normalized to $S(b,\vec{g},H(r)){|}_{b=0}$ according to Equation (A1). Below, we outline the key parameter and implementation choices:

- We assumed $S_{\text{e}}(b,\vec{g})=0$ for both in-vivo and ex-vivo dMRI. Although $S_{\text{e}}(b,\vec{g})=0$ , the extra-axonal compartment still influences $S(b,\vec{g},H(r))$ as $f_{\text{e}}>0$ .
- We assumed $f_{\text{im}}=0$ for in-vivo dMRI, assuming negligible signal contribution (Tax et al., 2020).
- We assumed $f_{\text{im}}=0.27$ for ex-vivo dMRI, reflecting the mean value in our experimental dMRI data.
- For $S_{\text{a}}^{⊥}(b,\xi (\vec{g},\vec{n}),r)$ , we used the matrix method (Callaghan, 1997). In particular, we used the implementation of the MISST toolbox (Drobnjak et al., 2011; Ianuş et al., 2016) to determine the relevant matrices and computed $S_{\text{a}}^{⊥}(b,\xi (\vec{g},\vec{n}),r)$ using an equation for rectangular waveform (see equation 26 in Callaghan, 1997). The latter implies the assumption of infinite slew rate.
- For $S_{\text{a}}^{∥}(b,\xi (\vec{g},\vec{n}))$ , we used fixed values from the literature (Veraart et al., 2018, 2020) (see Table 3).
- We assumed a single fiber bundle with fixed mean orientation $\vec{\mu }={\left[0,0,1\right]}^{T}$ and Watson-distributed fibers with orientation $\vec{n}$ (Watson, 1965).
- We numerically approximated the integral over $\vec{n}$ using Lebedev quadrature of degree 590 (Lebedev & Laikov, 1999; Parrish, 2024).
- We discretized r using anisotropic binning with edges ∈{0,0.1,...,5,5.2,...,10,10.5,...20} .
- The full set of simulation parameters is listed in Table 3.
