# Supplementary material for: MRI-scale histology validates spatial sensitivity of in-vivo MRI-based axon radius estimation
Source: Imaging Neurosci (Camb). 2025 Dec 3;3:IMAG.a.1030. doi: 10.1162/IMAG.a.1030 (PMC13288500; doi:10.1162/IMAG.a.1030)
Supplement: Supplementary Material [file IMAG.a.1030_supp.pdf]

Supplementary material for: MRI-scale histology validates spatial sensitivity of in-vivo MRI-based axon radius estimation

Laurin Mordhorst[1,2*], Luke J. Edwards[3,4], Maria Morozova[4,5], Mohammad Ashtarayeh[2], Tobias Streubel[2], Björn Fricke[1,2], Francisco J. Fritz[1,2], Henriette Rusch[5], Carsten Jäger[4,5], Ludger Starke[6], Thomas Gladytz[6], Ehsan Tasbihi[6,7], Joao S. Periquito[6], Andreas Pohlmann[6], Herbert Mushumba[8], Klaus Püschel[8], Thoralf Niendorf[6,7], Nikolaus Weiskopf[4,9,10], Markus Morawski[4,5], Siawoosh Mohammadi[1,2,11*]

[1]Department of Neuroradiology, University Hospital of Luebeck, Luebeck, Germany.

[2]Institute of Systems Neuroscience, University Medical Center Hamburg-Eppendorf, Hamburg, Germany.

[3]Department of Cognitive Neuroscience, Faculty of Psychology and Neuroscience,
Maastricht University, Maastricht, the Netherlands.

[4]Department of Neurophysics, Max Planck Institute for Human Cognitive and Brain Sciences, Leipzig, Germany.

[5]Paul Flechsig Institute - Centre of Neuropathology and Brain Research, Leipzig, Germany.

[6]Berlin Ultrahigh Field Facility (B.U.F.F.), Max Delbrück Center for Molecular Medicine
in the Helmholtz Association, Berlin, Germany.

[7]Charité – Universitätsmedizin Berlin, Berlin, Germany.

[8]Institute of Legal Medicine, University Medical Center Hamburg-Eppendorf, Hamburg, Germany.

[9]Felix Bloch Institute for Solid State Physics, Leipzig University, Leipzig, Germany.

[10]Wellcome Centre for Human Neuroimaging, Institute of Neurology,
University College London, London, United Kingdom.

[11]Max Planck Research Group MRI Physics, Max Planck Institute for Human Development, Berlin, Germany.

*Correspondence:laurin.mordhorst@uni-luebeck.de, siawoosh.nathomohammadi@uni-luebeck.de

October 26, 2025

S1 Impact of spatial smoothing on in-vivo dMRI-based r_{eff}

In the main manuscript, we applied spatial smoothing to in-vivo dMRI-based r_{eff} maps prior to comparison with histological values (see Figure 4g and Figure 5g). In Figure S1.1, we illustrate the impact of spatial smoothing on r_{eff}, including both a spatial r_{eff} pattern for an exemplary subject in native space (see Figure S1.1a-b) and its effect on the quantitative comparison with histology for the group-average r_{eff} (see Figure S1.1c-d).

Smoothing preserves the overall spatial pattern, maintaining the high-low trend across the genu, anterior midbody, midbody, posterior midbody, and splenium (see Figure S1.1a-b). However, it reduces local variations, making the pattern less sensitive to noise but potentially also to finer structural details. The quantitative comparison with histology in Figure S1.1c-d confirms that smoothing improves correlation. Importantly, smoothing appears to enhance precision without introducing spurious correlations.

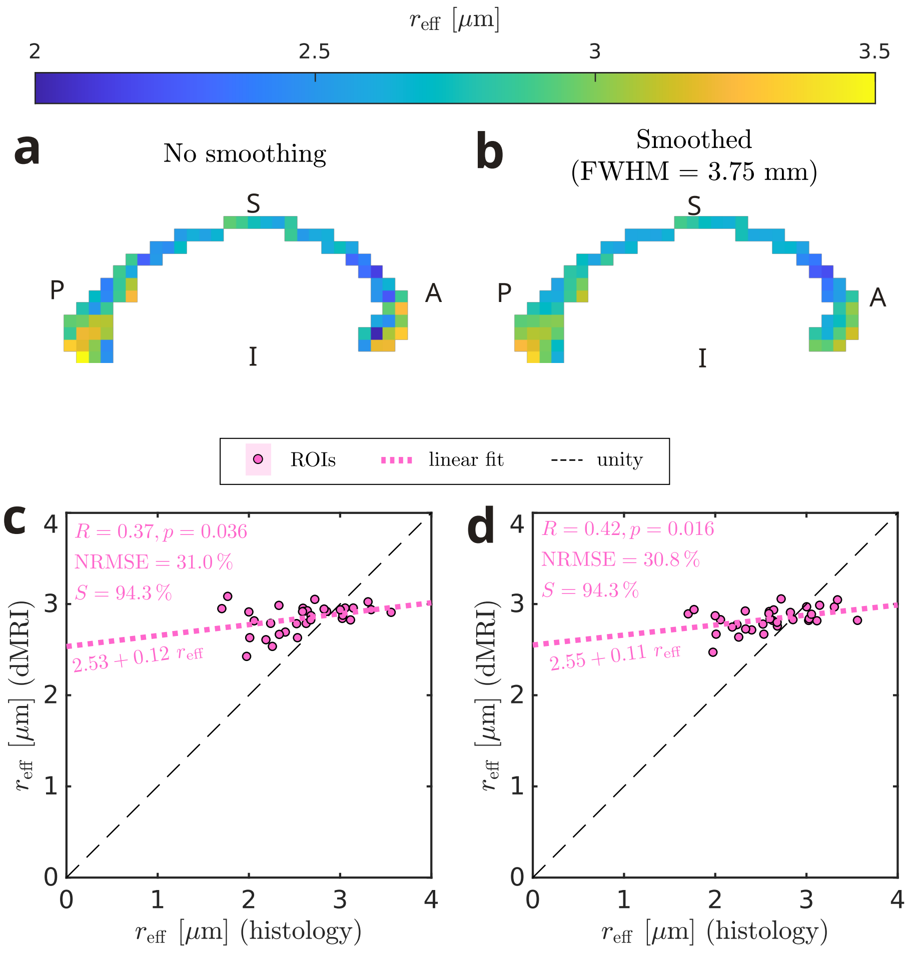

Figure S1.1: **Impact of spatial smoothing on in-vivo dMRI-based r_{eff}. (a-b)** Spatial patterns of r_{eff} across the corpus callosum: **(a)** before smoothing and **(b)** after smoothing with FWHM $= 3.75\,\text{mm}$ ($1.5\times$ voxel size). Patterns show mid-sagittal section of the corpus callosum for an exemplary subject in native space. **(c-d)** Quantitative comparison of in-vivo dMRI-based group-average r_{eff} against histology. The group-average r_{eff} was computed from different per-subject maps: **(c)** from unsmoothed maps, as illustrated in (a), and **(d)** from smoothed maps, as illustrated in (b). Markers represent histological ROIs in Figure 1a. The dashed lines illustrate theoretical perfect agreement. Annotations provide metrics computed over all ROIs, including Pearson's correlation coefficient (R) and the corresponding p-value, the normalized root-mean-square error (NRMSE), and the fitting success rate (S) (see Equations (6) to (9)).

S2 Influence of Gaussian and Rician noise on r_{eff} estimation

We used Rician-distributed magnitude dMRI for our experimental validation of r_{eff}, shown in Figure 5a. To mimic these conditions, we also incorporated Rician noise into our dMRI simulations (see Figure 5b). For comparison, we replicated these simulations using Gaussian noise to assess the potential improvements achievable with Gaussian-distributed signals, assuming such distributions can be achieved through advanced preprocessing techniques (Eichner et al., 2015; Fan et al., 2020; Manzano Patron et al., 2024).

Figure S2.1a-b compare the impact of Gaussian and Rician noise on r_{eff} from dMRI simulations against histological r_{eff}, both for the ex-vivo and in-vivo scenario. In ex-vivo dMRI simulations (see Figure S2.1a), the noise distribution has little impact, likely due to the large number of diffusion measurements (585 directions across shells). In-vivo (see Figure S2.1b), Gaussian noise allows for better precision than Rician noise, translating into improved R and NRMSE. It should be noted that the impact of Rician noise on r_{eff} estimates is likely underestimated here, as the noise level σ was assumed to be known in our simulations. In practice, σ is typically unknown and must be estimated. This σ-estimation, likely imperfect, can be expected to propagate additional errors into r_{eff}.

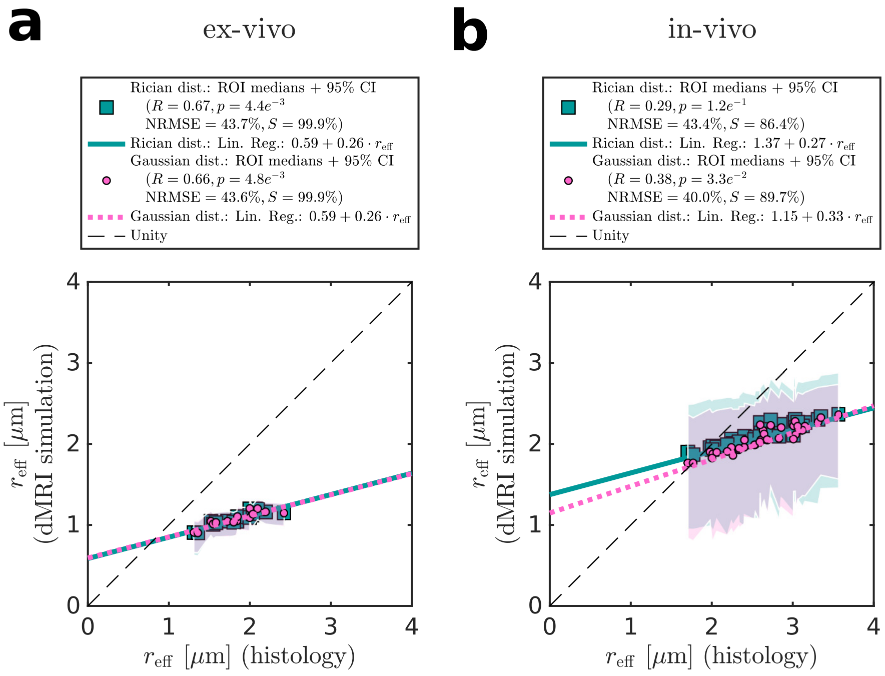

Figure S2.1: **Influence of Gaussian and Rician noise on r_{eff} estimation.** Comparison of r_{eff} from dMRI simulations against histological values for our experimental **(a)** ex-vivo and **(a)** in-vivo dMRI protocols. Results are shown for both Gaussian and Rician noise (see legend). Markers represent histological ROIs in Figure 1a. The $95\,\%$ confidence intervals (shaded areas) were computed across 1000 noise realizations. The dashed lines illustrate theoretical perfect agreement. The legends provide metrics computed over all ROIs, including Pearson's correlation coefficient (R) and the corresponding p-value, the normalized root-mean-square error (NRMSE), and the fitting success rate (S) (see Equations (6) to (9)).

S3 Axon radius distributions from light microscopy

Figure S3.1 shows axon radius distributions from our two histological tissue samples for five regions of the corpus callosum: genu, anterior midbody, midbody, posterior midbody, and splenium. Axon radius distributions and r_{eff} are similar across tissue samples (see Figure S3.1c-f), except for the genu (see Figure S3.1b). Yet, tissue sample CC-02 generally exhibits slightly higher r_{eff} than CC-01. The pattern of r_{eff} across anterior midbody, midbody, posterior midbody, and splenium (see Figure S3.1c-f) follows an alternating low-high pattern.

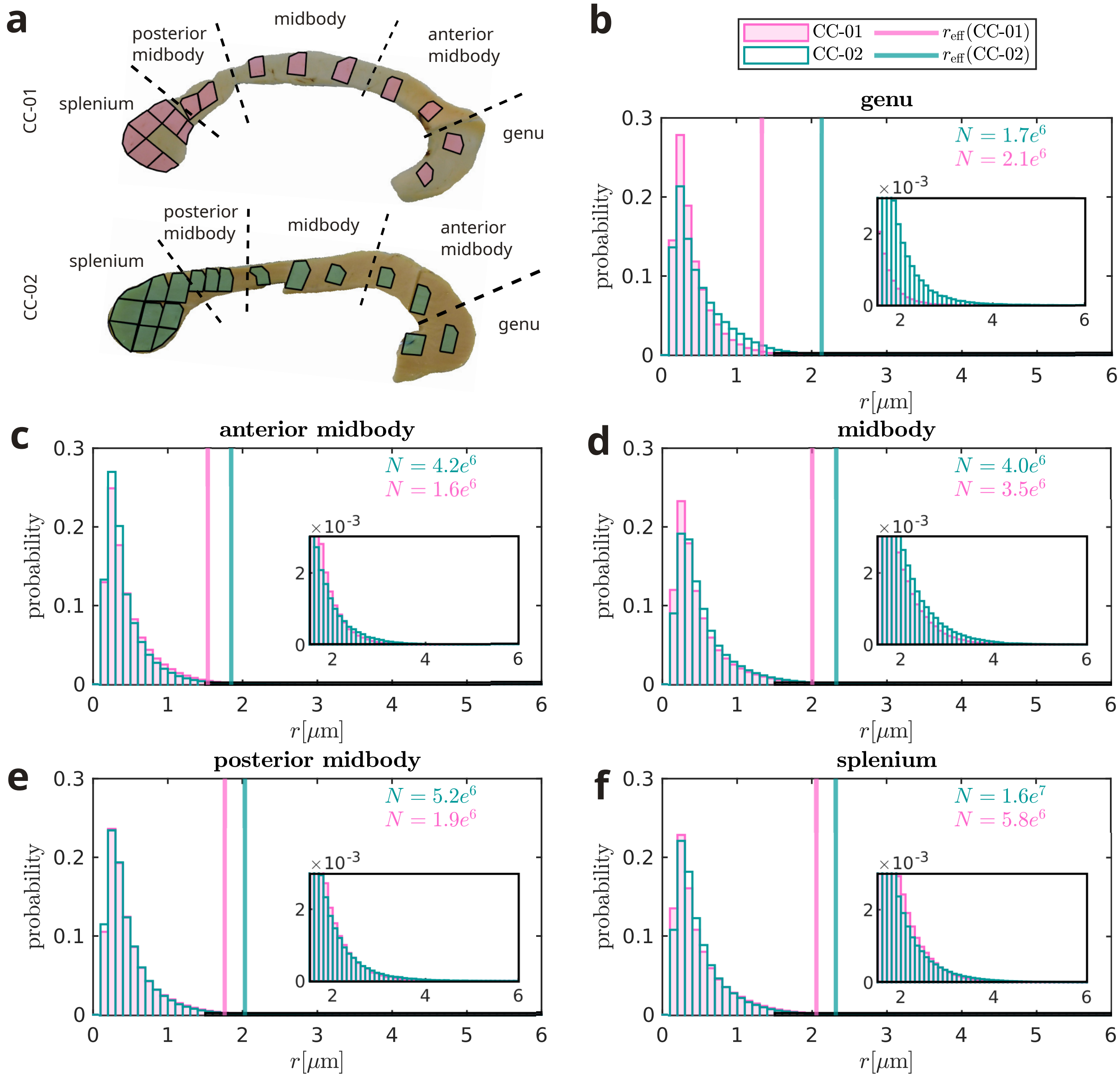

Figure S3.1: **Axon radius distributions from light microscopy. (a)** Corpus callosum sketch with light microscopy ROIs (polygons) and segmentation into five regions (dashed lines). **(b-f)** Axon radius distributions pooled over the light microscopy ROIs for each corpus callosum region shown in (a). Colors indicate tissue sample (see legend in (b)). Vertical lines denote r_{eff} as per Equation (1). Inter-donor differences across subregions in (b-f) were on average $0.39\,\mu\text{m}$ ($21\,\%$ relative to the mean across subregions and donors) with a standard deviation of $0.23\,\mu\text{m}$ ($14\,\%$). Annotated numbers inside the plot denote axon count. Insets highlight the tail of axon radius distributions.

S4 Reproducibility of correlation between in-vivo dMRI-based r_{eff} and histological r_{eff}

In-vivo dMRI-based r_{eff} exhibited some variability due to the non-deterministic nature of our processing pipeline. The non-determinism arose from the GPU-accelerated slice-to-volume eddy current and motion correction (Andersson & Sotiropoulos, 2016; Andersson et al., 2016; Tournier et al., 2019), which is currently only implemented on GPU. This step introduced slight variability in geometric corrections between runs that propagated into subsequent processing stages and ultimately affected voxel-wise r_{eff} correspondences with histological values. To assess the impact of this variability on correlation, we repeated the quantitative analysis in Figure 5a for ten separate runs of the pipeline.

Qualitatively, comparison group-average r_{eff} from in-vivo dMRI show a consistent trend across all iterations. Pearson's R varied between 0.37 ($p = 0.032$, see Figure S4.1h) to 0.47 ($p < 0.006$, see Figure S4.1a,g), with a mean of $R = 0.42 \pm 0.03$. All correlations were statistically significant ($p < 0.05$).

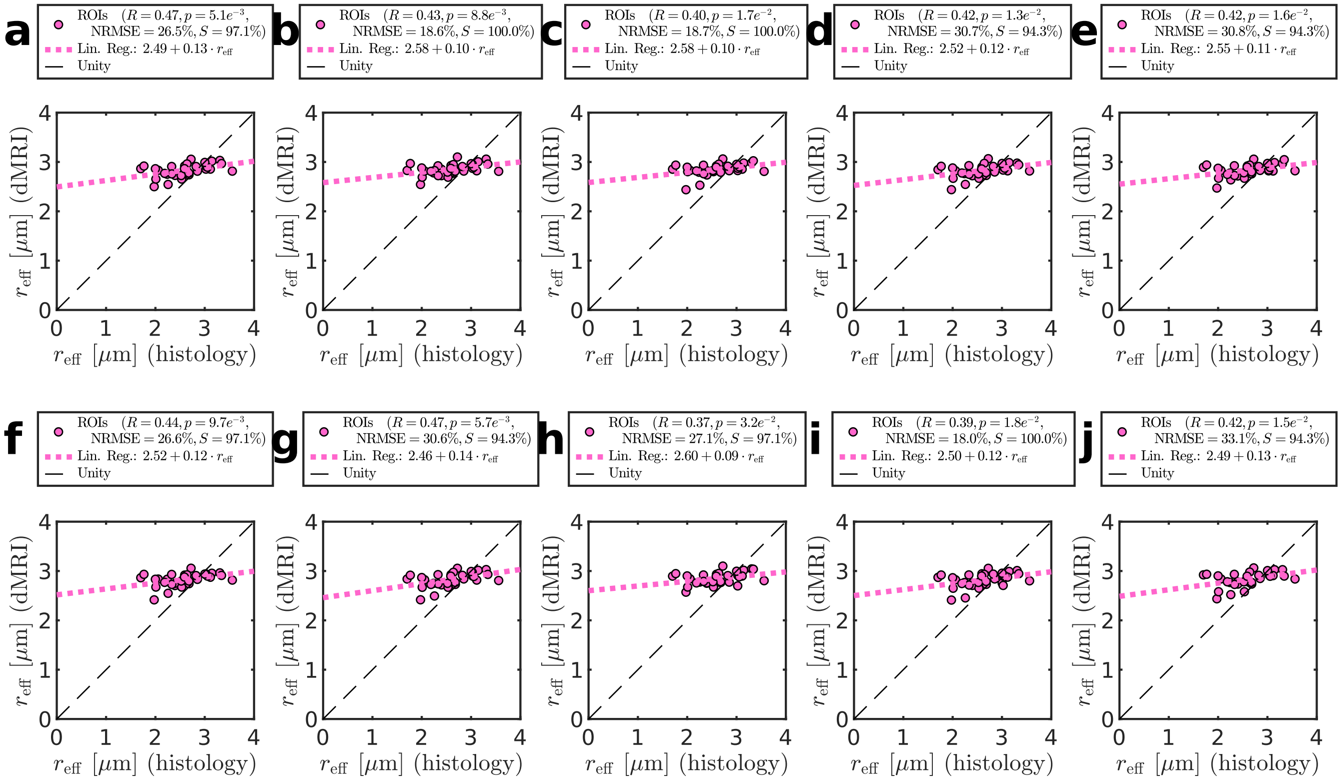

Figure S4.1: **Reproducibility of correlation between in-vivo dMRI-based r_{eff} and histological r_{eff}. (a-j)** Quantitative comparisons of r_{eff} from in-vivo dMRI against histology. Each plot represent one of ten repeated runs through our in-vivo processing pipeline. Markers represent histological ROIs from Figure 1a, with in-vivo dMRI-based r_{eff} reflecting the group-average. The dashed lines illustrate theoretical perfect agreement. The legends provide metrics computed over all ROIs, including Pearson's correlation coefficient (R) with corresponding p-value, the normalized root-mean-square error (NRMSE) , and the fitting success rate (S) (see Equations (6) to (9)).

S5 Per-subject analyses for in-vivo dMRI-based r_{eff}

While Figure 4g and Figure 5a in the main manuscript presents group-average r_{eff} for our in-vivo dMRI experiments, here we show per-subject results, including both spatial patterns across the corpus callosum (see Figure S5.1a-e) and quantitative comparisons with histology (see Figure S5.1f-j).

The spatial r_{eff} patterns are consistent across subjects in showing higher values in the genu and splenium and lower values in the anterior midbody. However, overall, inter-subject variability appears to be substantial. This variability is also reflected in the quantitative comparisons with histology: while r_{eff} generally increases with histological values for most subjects (see Figure S5.1f-i), there's a significant correlation with histology only for one subject (see Figure S5.1g).

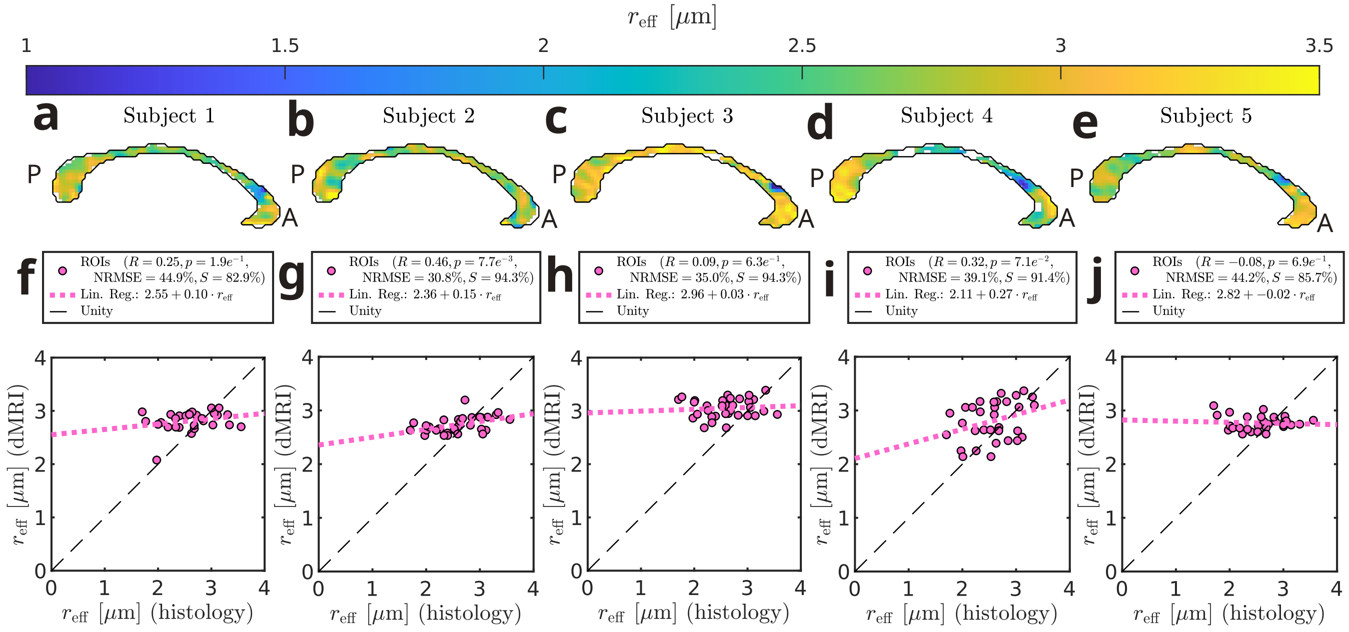

Figure S5.1: **Per-subject analyses for in-vivo dMRI-based r_{eff}. (a-e)** Per-subject spatial patterns of r_{eff} across the corpus callosum, shown in mid-sagittal section of the Oxford-MultiModal-1 (OMM-1, Arthofer et al., 2024) atlas space. **(f-j)** Per-subject quantitative comparison of in-vivo dMRI-based r_{eff} against histology. Markers represent histological ROIs in Figure 1a. The dashed lines illustrate theoretical perfect agreement. The legends provide metrics computed over all ROIs, including Pearson's correlation coefficient (R) and the corresponding p-value, the normalized root-mean-square error (NRMSE), and the fitting success rate (S) (see Equations (6) to (9)).

S6 Covariation of f_{im} and ex-vivo dMRI-based r_{eff}

For ex-vivo dMRI experiments, we estimated f_{im} in a separate step prior to r_{eff} estimation (see Section 3.2.4). Here, we assessed whether ex-vivo r_{eff} estimates covary with f_{im}, as such covariation would indicate a potential confounding dependency in r_{eff} estimation. Figure S6.1 illustrates how this estimation affects r_{eff}, showing both spatial patterns (see Figure S6.1a-b) and the relationship between r_{eff} and f_{im} (see Figure S6.1c).

The spatial pattern of r_{eff} suggest a relationship with f_{im}. This relationship is particularly evident in the splenium, where sampling density is highest. The quantitative comparison in Figure S6.1c further supports this by showing that r_{eff} scales approximately linearly with f_{im} in the splenium. These findings indicate that f_{im} is a confounding factor in r_{eff} estimation, at least for our f_{im} estimation approach.

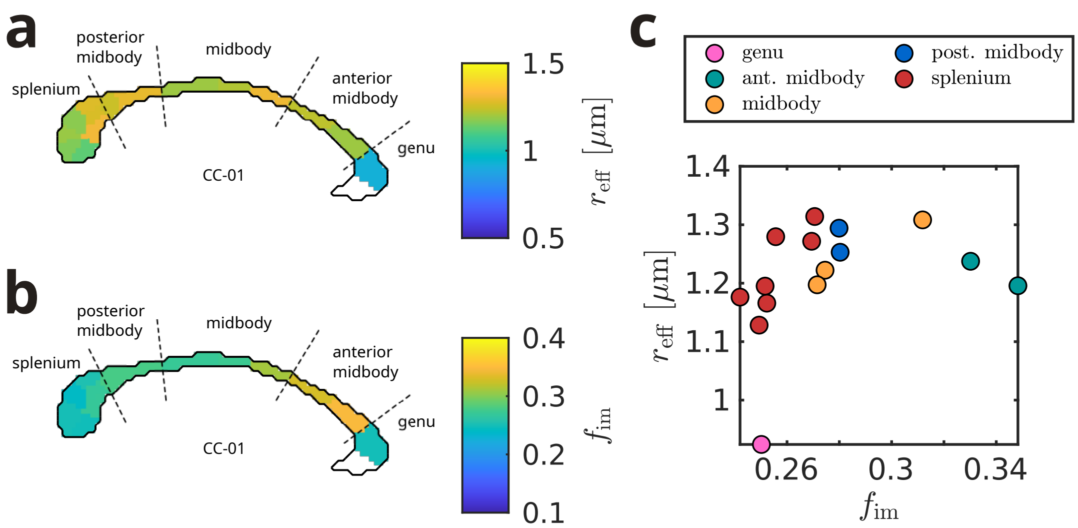

Figure S6.1: **Impact of f_{im} on ex-vivo dMRI-based r_{eff} (a)** Spatial patterns of ex-vivo dMRI-based r_{eff} across the corpus callosum, shown in mid-sagittal Oxford-MultiModal-1 (OMM-1, Arthofer et al., 2024) atlas slice with subregions indicated (dashed lines). Spatial patterns were interpolated using the nearest-neighbor method, evaluated at the ROI locations in Figure 1a. The void in the genu indicates a ROI not scanned with ex-vivo dMRI (see Figure 2e). **(b)** Corresponding spatial pattern of f_{im}. **(c)** Relationship between f_{im} and r_{eff}. Each marker represents one ROI from Figure 1a, with marker colors indicating corpus callosum subregions as defined by dashed lines in (a-b).

S7 Impact of f_{im} variation

dMRI-based r_{eff} estimation requires an assumption about the immobile water fraction f_{im} (see Equation (2)). For ex-vivo data, we estimated f_{im} prior to r_{eff} fitting, whereas for in-vivo data we assumed $f_{\mathrm{im}} = 0$. Here, we tested how deviations from these assumed values affect r_{eff} by simulating signals with variable underlying f_{im}. To this end, we kept the assumed values fixed for fitting ($f_{\mathrm{im}} = 0.27$ ex-vivo and $f_{\mathrm{im}} = 0$ in-vivo), while the true f_{im} used for signal generation was randomly sampled from a lognormal distribution to enforce positive f_{im}. The distribution was parameterized by a mean $\mu = 0.27$ ex-vivo and $\mu = 0.002$ in-vivo (Tax et al., 2020), with variable coefficient of variation (CV). Corresponding lognormal parameters were $\sigma_\ell^2 = \ln(1 + \mathrm{CV}^2)$ and $\mu_\ell = \ln(\mu) - \frac{1}{2}\sigma_\ell^2$.

Figure S7.1 illustrates the impact of f_{im} variation on r_{eff} under ex-vivo conditions. Even for low variability ($\mathrm{CV} = 2\,\%$; see Figure S7.1a), the correlation is already noticeably degraded compared to simulations with perfectly known f_{im} ($R = 0.35$ versus $R = 0.55$, cf. Figure 5b). At higher variability ($\mathrm{CV} = 5\,\%$ or $10\,\%$; see Figure S7.1b-c), the correlations are further degraded, highlighting that r_{eff} estimates are highly sensitive to uncertainty in f_{im} – at least under the low sensitivity of the ex-vivo dMRI protocol investigated here.

Figure S7.2 shows the impact of f_{im} variability on r_{eff} under in-vivo conditions, using the optimized next-generation clinical scanner protocols across different SNR levels defined in Figure 8c-e. We report only the highest variability tested in the ex-vivo analysis (CV $= 10\,\%$), as lower levels had negligible effect. Even in this case, the impact on correlation remains modest compared to simulations with $f_{\mathrm{im}} = 0$ in Figure 8c-e in the main manuscript ($R = 0.27$ versus $R = 0.29$ at SNR ≈ 27; $R = 0.47$ versus $R = 0.49$ at SNR ≈ 48; $R = 0.60$ versus $R = 0.63$ at SNR ≈ 68). This indicates that in-vivo r_{eff} estimates are robust to uncertainty in f_{im} and that the small $f_{\mathrm{im}} = 0.002$ reported by Tax et al. (2020) can safely be neglected.

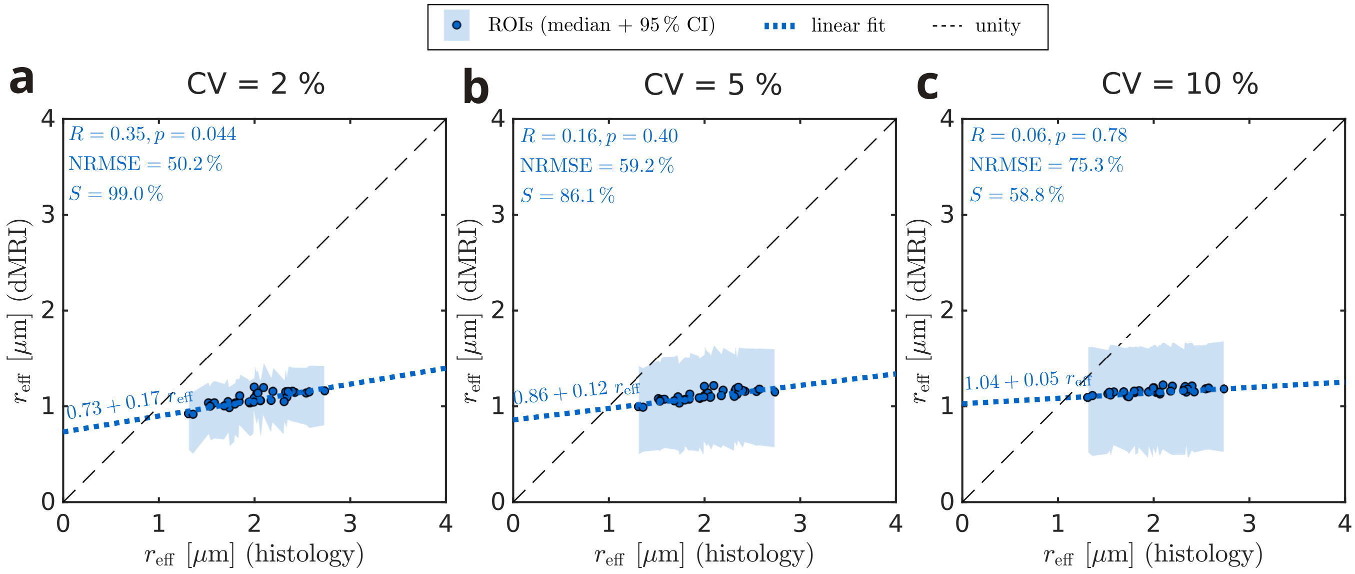

Figure S7.1: **Impact of f_{im} variation on ex-vivo r_{eff} mapping. (a–c)** Comparison of simulated r_{eff} with histological values for the experimental ex-vivo dMRI protocol. In the simulations, true f_{im} values were randomly sampled from a lognormal distribution with mean $\mu = 0.27$ and different coefficients of variation (CoV, see row titles), while a fixed $f_{\mathrm{im}} = 0.27$ was assumed for fitting. Markers represent histological ROIs in Figure 1a. The $95\,\%$ confidence intervals (shaded areas) were computed across 1000 noise realizations. The dashed lines illustrate theoretical perfect agreement. Annotated metrics were computed over all ROIs, including Pearson's correlation coefficient (R) and the corresponding p-value, the normalized root-mean-square error (NRMSE), and the fitting success rate (S) (see Equations (6) to (9)).

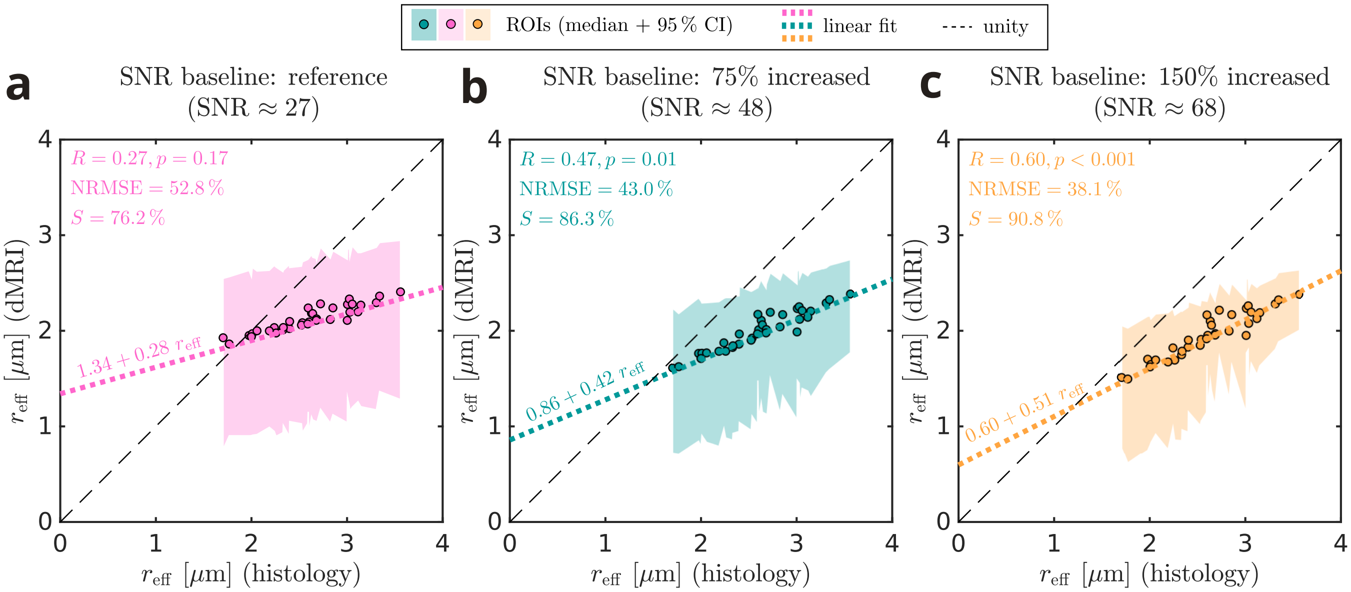

Figure S7.2: **Impact of f_{im} variation on in-vivo r_{eff} mapping. (a–c)** Comparison of simulated r_{eff} with histological values for the optimized next-generation clinical scanner protocols across different baseline SNR levels. In the simulations, true f_{im} values were randomly sampled from a lognormal distribution with mean $\mu = 0.002$ (as reported by Tax et al. (2020)) and fixed coefficient of variation $\text{CV} = 10\,\%$, while $f_{\text{im}} = 0$ was assumed for fitting. Markers represent histological ROIs in Figure 1a. The $95\,\%$ confidence intervals (shaded areas) were computed across 1000 noise realizations. The dashed lines illustrate theoretical perfect agreement. Annotated metrics were computed over all ROIs, including Pearson's correlation coefficient (R) and the corresponding p-value, the normalized root-mean-square error (NRMSE), and the fitting success rate (S) (see Equations (6) to (9)).

S8 dMRI protocol optimization (additional results)

In the main manuscript, we optimized in-vivo dMRI protocols assuming a radius scaling factor $\eta = 1.3$ for tissue shrinkage compensation (Aboitiz et al., 1992; Tang et al., 1997). Here we repeated in-vivo protocol optimization for $\eta = 1.0$ and $\eta = 1.5$, representing more extreme scenarios of no tissue shrinkage and very strong tissue shrinkage. Following the approach in the main manuscript, we conducted the protocol optimization analyses with increased baseline SNR levels to study the potential impact of technical or acquisition improvements.

Figure S8.1 shows R and NRMSE as a function of $g_{\max}$ for different η. For any η, R curves show similar qualitative behavior, converging to a maximum R for a certain $g_{\max}$ (see Figure S8.1a-c), roughly coinciding with optimal NRMSE (see Figure S8.1d-f). Yet, for $\eta = 1$, this $g_{\max}$ is outside the range of values of exisiting in-vivo scanners at reference SNR baseline level (see Figure S8.1a). For $\eta \geq 1.3$ optimal R can be achieved with existing scanners (see Figure S8.1b-c), with NRMSE becoming suboptimal (larger) for higher $g_{\max}$ (see Figure S8.1e-f).

Figure S8.2 shows comparisons of simulated r_{eff} estimates against histological values for the optimal protocols across different scanners, complementing the metrics shown in Figure S8.1. These plots illustrate that R and NRMSE are determined by precision rather than bias for small η and $g_{\max}$. Yet, at large η and $g_{\max}$, the model-inherent bias strongly reduces the sensitivity to large axons, leading to the non-optimal NRMSE at high $g_{\max}$ values observed in Figure S8.1e-f.

Table S8.1 show the optimal parameters for protocols presented in Figures S8.1 and S8.2. The parameters for $\eta = 1.3$ correspond to the optimal parameters shown in (see Figure 8).

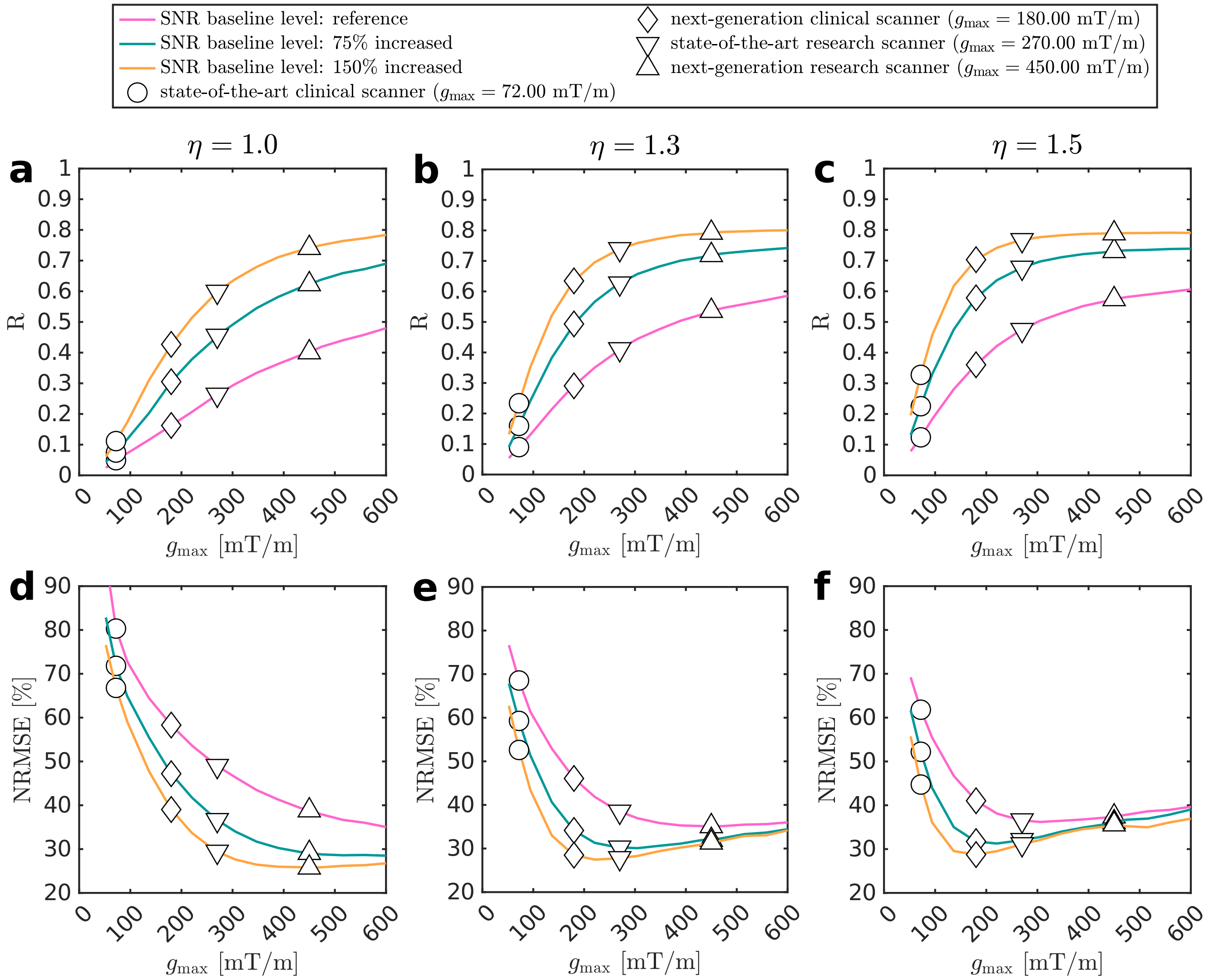

Figure S8.1: **Optimal in-vivo protocol metrics for different axon radius scaling factors. (a-c)** Optimal Pearson's correlation coefficients (R) as a function of maximum gradient amplitude ($g_{\max}$). Columns show R for different radius scaling factors used to compensate for tissue shrinkage: **(a)** $\eta = 1$, **(b)** $\eta = 1.3$ and **(c)** $\eta = 1.5$. While $\eta = 1.3$ (Aboitiz et al., 1992; Tang et al., 1997) was used as an estimate for in-vivo conditions in the main manuscript, $\eta = 1.0$ and $\eta = 1.5$ represent the scenarios of no tissue shrinkage and very strong tissue shrinkage. Markers encode $g_{\max}$ of existing clinical scanners and research scanners (assuming $90\,\%$ of the nominal $g_{\max}$). Line colors indicate different SNR baseline levels. While the reference SNR baseline level reflects our experimental conditions, increased SNR baseline levels assume an SNR increase through potential technical or acquisition advances. In addition, the SNR of a particular protocol candidate depends on the protocol parameters (see Equation (13)). For our experimental protocol, baseline SNR levels would correspond to SNR values of 32 (reference), 56 ($75\,\%$ increased) and 80 ($150\,\%$ increased). **(d-f)** Optimal normalized root-mean-square error (NRMSE) as a function of maximum gradient amplitude ($g_{\max}$) for different η, with definitions as in (a-c). Note that we optimized protocols by maximizing R, whereas NRMSE is an auxiliary metric.

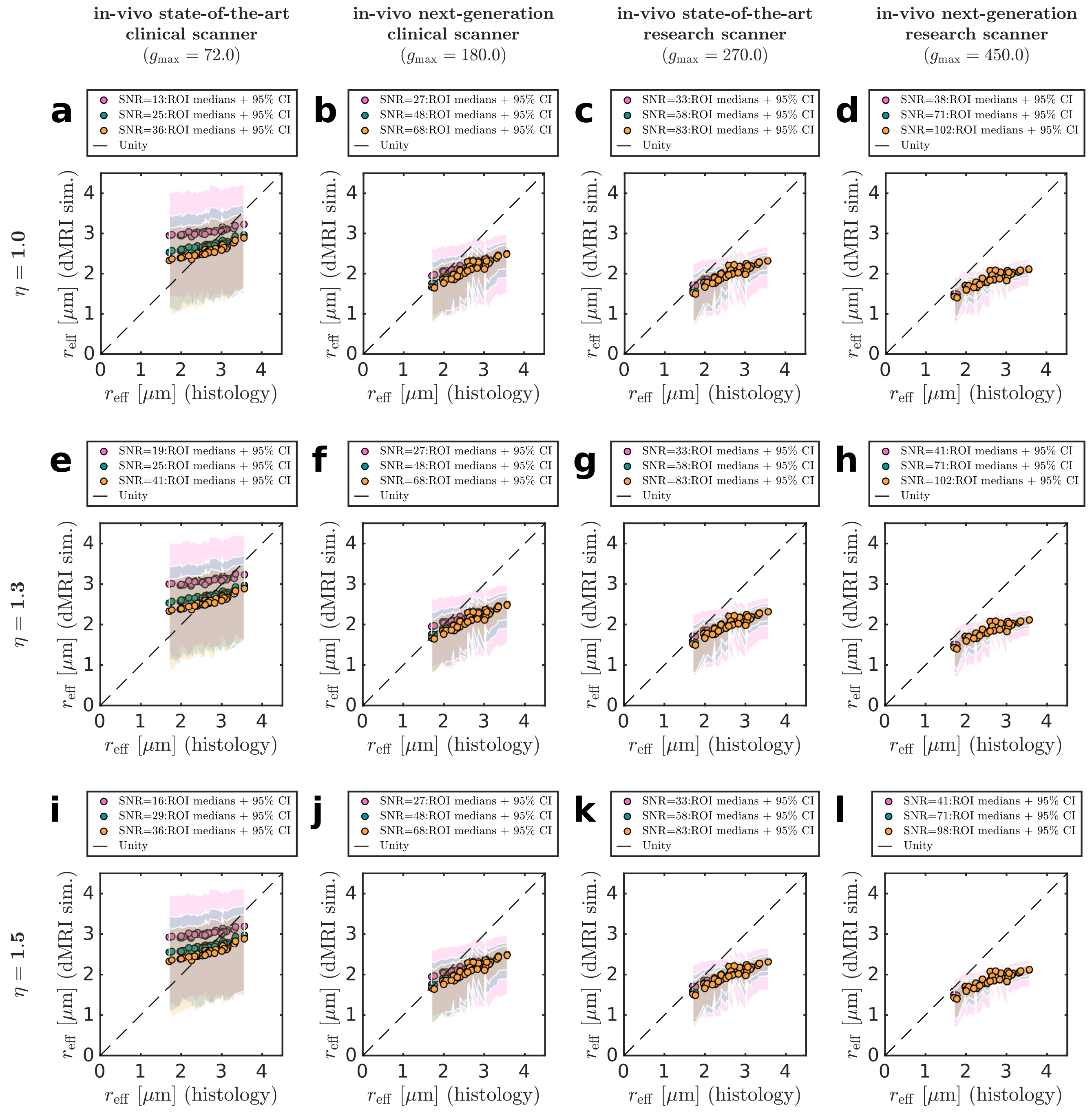

Figure S8.2: **Estimated r_{eff} from optimal in-vivo dMRI protocols.** The plots show r_{eff} derived from dMRI simulations with optimal protocols against the histological r_{eff} for various maximum gradient amplitudes g_{max} of existing/upcoming scanners (columns), assuming $90\,\%$ of nominal values. The η values (rows) denote radius scaling factors compensating for tissue shrinkage in histology data. While $\eta = 1.3$ reflects the value assumed in the main manuscript, $\eta = 1$ and $\eta = 1.5$ reflect more extreme cases of no tissue shrinkage or very strong tissue shrinkage. Markers represent histological ROIs in Figure 1a. The $95\,\%$ confidence interval for ROIs (shaded areas) was computed across 1000 noise realizations. Colors indicate different SNR baseline levels. While the reference SNR baseline level reflects our experimental conditions, increased SNR baseline levels assume an SNR increase through potential technical or acquisition advances. In addition, the SNR of a particular protocol candidate depends on the protocol parameters (see Equation (13)). See Table S8.1 for the full set of protocol parameters and metrics for protocols shown here.

Table S8.1: **Parameters of optimal in-vivo dMRI protocols.** All dMRI protocols use two shells with $\{60, 120\}$ diffusion gradient directions. The SNR baseline level describes the SNR conditions compared to our dMRI experiments. While the reference SNR baseline level corresponds to our experimental conditions, increased SNR baseline levels assume an SNR increase through potential technical or acquisition advances. η is a radius scaling factor compensating for tissue shrinkage in histology data, where $\eta = 1.3$ reflects the value assumed in the main manuscript. $g_{\min}$ and $g_{\max}$ are the diffusion gradient amplitudes of the two diffusion shells; $b_{\min} = 6\,\mathrm{ms}/\mu\mathrm{m}^2$ and $b_{\max}$ denote the corresponding diffusion-weightings. δ and Δ are the gradient diffusion time and separation, consistent across shells. Echo time (T_E) was estimated according to Equation (11). The SNR was estimated using Equation (13), reflecting not only the baseline SNR level, but also protocol parameter-dependent SNR variation. Pearson's correlation coefficient (R) and its associated p-value, as well as the normalized root-mean square-error (NRMSE), and the fitting success rate (S) were computed using Equations (6) to (9). The optimal dMRI protocol with maximum R per combination of η and SNR baseline level is highlighted in bold.

SNR baseline	η	$g_{\max}$ [mT/m]	$g_{\min}$ [mT/m]	$b_{\max}$ [ms/μm^2]	δ [ms]	Δ [ms]	T_E [ms]	SNR	R	p	NRMSE [%]	S [%]
reference	1.0	72	30.9	32.5	48	54	124	12.8	0.05	$8.4e^{-1}$	80.3	54.2
		180	81.3	29.4	24	30	76	27.3	0.16	$4.5e^{-1}$	58.3	68.7
		270	119.9	30.4	18	24	64	33.2	0.26	$1.8e^{-1}$	49.0	78.4
		**450**	**167.0**	**43.6**	**14**	**20**	**56**	**38.0**	**0.40**	$2.5e^{-2}$	**38.8**	**89.6**
	1.3	72	46.4	14.4	36	42	100	18.6	0.09	$7.0e^{-1}$	68.5	59.2
		180	81.3	29.4	24	30	76	27.3	0.29	$1.3e^{-1}$	46.1	82.5
		270	119.9	30.4	18	24	64	33.2	0.41	$2.0e^{-2}$	38.5	91.4
		**450**	**203.9**	**29.2**	**12**	**18**	**52**	**40.7**	**0.54**	$< 1e^{-3}$	**35.1**	**97.5**
	1.5	72	40.1	19.4	40	46	108	16.4	0.12	$5.8e^{-1}$	61.8	65.3
		180	81.3	29.4	24	30	76	27.3	0.36	$4.6e^{-2}$	41.0	89.4
		270	119.9	30.4	18	24	64	33.2	0.47	$4.8e^{-3}$	36.6	96.0
		**450**	**203.9**	**29.2**	**12**	**18**	**52**	**40.7**	**0.57**	$< 1e^{-3}$	**37.4**	**99.2**
75% increased	1.0	72	35.0	25.4	44	50	116	25.3	0.07	$7.6e^{-1}$	71.8	57.9
		180	81.3	29.4	24	30	76	47.7	0.31	$1.2e^{-1}$	47.2	79.3
		270	119.9	30.4	18	24	64	58.1	0.46	$9.9e^{-3}$	36.6	89.1
		**450**	**203.9**	**29.2**	**12**	**18**	**52**	**71.1**	**0.62**	$< 1e^{-3}$	**29.1**	**96.6**
	1.3	72	35.0	25.4	44	50	116	25.3	0.16	$4.6e^{-1}$	59.3	67.7
		180	81.3	29.4	24	30	76	47.7	0.49	$3.7e^{-3}$	34.1	92.7
		270	119.9	30.4	18	24	64	58.1	0.63	$< 1e^{-3}$	30.2	97.6
		**450**	**203.9**	**29.2**	**12**	**18**	**52**	**71.1**	**0.72**	$< 1e^{-3}$	**32.0**	**99.7**
	1.5	72	40.1	19.4	40	46	108	28.7	0.23	$2.7e^{-1}$	52.2	75.1
		180	81.3	29.4	24	30	76	47.7	0.58	$< 1e^{-3}$	31.7	96.9
		270	119.9	30.4	18	24	64	58.1	0.68	$< 1e^{-3}$	32.1	99.3
		**450**	**203.9**	**29.2**	**12**	**18**	**52**	**71.1**	**0.73**	$< 1e^{-3}$	**36.1**	**100.0**
150% increased	1.0	72	35.0	25.4	44	50	116	36.2	0.11	$6.3e^{-1}$	66.8	61.4
		180	81.3	29.4	24	30	76	68.1	0.43	$1.9e^{-2}$	39.0	86.1
		270	119.9	30.4	18	24	64	83.0	0.60	$< 1e^{-3}$	29.4	94.2
		**450**	**203.9**	**29.2**	**12**	**18**	**52**	**101.6**	**0.74**	$< 1e^{-3}$	**25.8**	**98.6**
	1.3	72	40.1	19.4	40	46	108	40.9	0.23	$2.5e^{-1}$	52.6	74.1
		180	81.3	29.4	24	30	76	68.1	0.63	$< 1e^{-3}$	28.5	96.6
		270	119.9	30.4	18	24	64	83.0	0.74	$< 1e^{-3}$	27.8	99.2
		**450**	**203.9**	**29.2**	**12**	**18**	**52**	**101.6**	**0.79**	$< 1e^{-3}$	**31.3**	**100.0**
	1.5	72	35.0	25.4	44	50	116	36.2	0.33	$8.5e^{-2}$	44.7	82.3
		180	81.3	29.4	24	30	76	68.1	0.70	$< 1e^{-3}$	28.8	98.8
		270	119.9	30.4	18	24	64	83.0	0.77	$< 1e^{-3}$	31.1	99.8
		**450**	**190.8**	**33.4**	**12**	**20**	**54**	**98.2**	**0.79**	$< 1e^{-3}$	**35.6**	**100.0**

S9 Impact of inter-cohort age differences

Axon radius has been reported to increase with age in human white matter (Aboitiz et al., 1996; Fan et al., 2019). This raises the question of whether the age mismatch between our in-vivo subjects (mean age: 31 years) and histological donors (mean age: 61 years) could affect correlations. To assess this, we repeated the correlation analysis for the optimal next-generation clinical scanner protocols in Figure 8c-e, but with histological radii extrapolated to a younger cohort (31 years). Specifically, we reduced histological radii by $5.3\,\%$, as estimated from the regression slope reported by Fan et al. (2019) for corpus callosum axon radii (their Figure 4a), corresponding to the shift from 61 to 31 years.

Figure S9.1 shows the corresponding comparisons of simulated in-vivo dMRI-based r_{eff} against histological values at different SNR levels. These plots are the age-corrected analog of Figure 8c-e. Compared to the uncorrected case, correlation coefficients (R) are slightly reduced compared to Figure 8c-e in the main manuscript ($R = 0.27$ versus $R = 0.29$ at SNR ≈ 27; $R = 0.45$ versus $R = 0.49$ at SNR ≈ 48; $R = 0.60$ versus $R = 0.63$ at SNR ≈ 68). Nonetheless, a significant moderate correlation could still be achieved at $\mathrm{SNR} \approx 48$ ($R = 0.45$, $p = 0.01$).

These results should be interpreted with caution. We parameterized the age effect using a dMRI study (Fan et al., 2019) with 36 subjects spanning a broad age range, enabling regression of age effects. However, we acknowledge that dMRI-derived radius estimates entail measurement uncertainties and potential biases, such as the model-inherent bias discussed in our study. Moreover, our simulations assumed a uniform reduction across all radii, which may be oversimplified (Aboitiz et al., 1996).

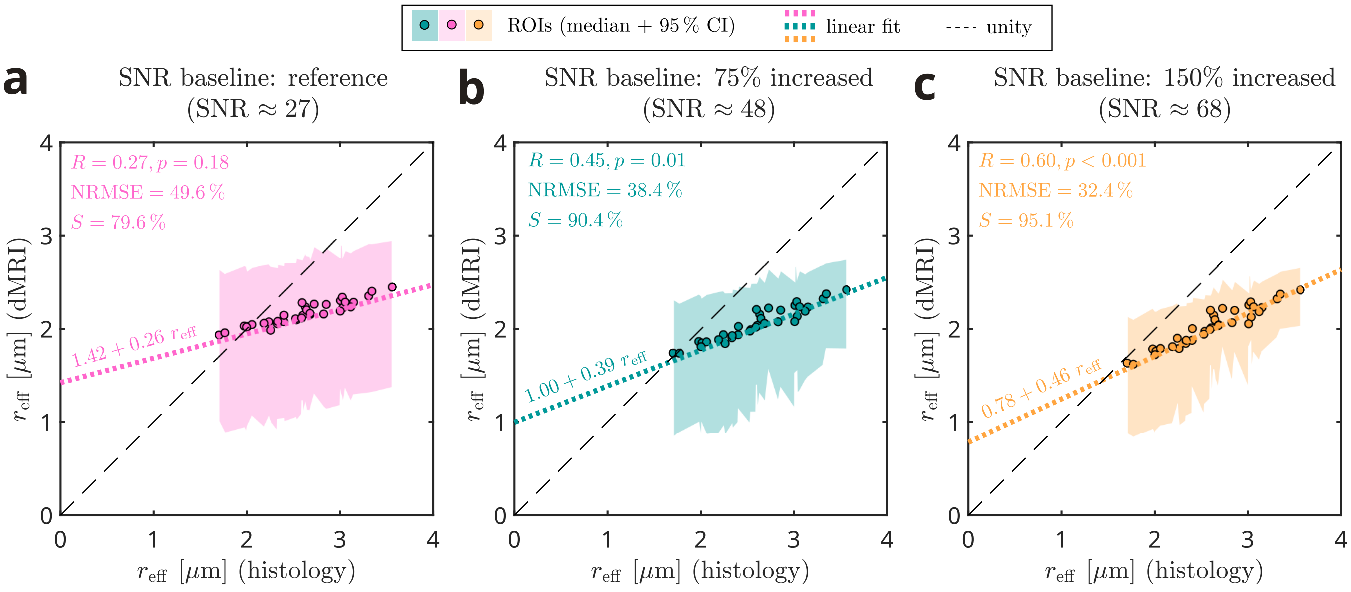

Figure S9.1: **Impact of inter-cohort age differences.** **(a-c)** Comparison of estimated r_{eff} with histological values for optimal next-generation clinical scanner protocols across baseline SNR levels. Here, histological radii were reduced by $5.3\,\%$ to approximate the shift from the histology donor age (61 years) to the in-vivo cohort age (31 years), based on the regression reported in Fan et al. (2019). SNR values of protocols are annotated above plots. Markers represent histological ROIs in Figure 1a. The $95\,\%$ confidence intervals (shaded areas) were computed across 1000 noise realizations. The dashed lines illustrate theoretical perfect agreement. The annotated metrics were computed over all ROIs, including Pearson's correlation coefficient (R) and the corresponding p-value, the normalized root-mean-square error (NRMSE), and the fitting success rate (S) (see Equations (6) to (9)).

S10 Simulation of clinical application for r_{eff} mapping

Motivated by reports of a $28.6\,\%$ reduction in axon radii in the splenium of individuals with autism spectrum disorder (ASD), we conducted a statistical power analysis to evaluate the feasibility of using r_{eff} to distinguish individuals with ASD from healthy controls. The analysis was conducted using the optimized protocols for next-generation clinical scanners. We estimated statistical power using a Monte Carlo simulation over $M = 5000$ iterations. We assumed $L = 11$ voxels in the splenium, reflecting the number of voxels in the mid-sagittal slice of the splenium in our in-vivo dMRI data. For various group sizes (N), we proceeded as follows:

- We randomly sampled $L \times N$ axon radius distributions of splenium ROIs from the histological dataset in Figure 1a per group (healthy and ASD), with replacement.
- For the ASD group, we scaled down each sampled axon radius by $28.6\,\%$ (see Figure S10.1a).
- For each sampled axon radius distribution, we simulated dMRI signals and estimated r_{eff}.
- We computed the mean r_{eff} across the L splenium voxels to obtain subject-level mean r_{eff} (see Figure S10.1b).
- We performed a two-sample t-test to assess group differences in subject-level mean r_{eff} with significance level $\alpha = 0.05$ (see Figure S10.1c).

Finally, based on the t-test results, we estimated the statistical power as the proportion of iterations that rejected the null hypothesis, which states that there is no difference between group means. This statistical power simulation design parallels prior work that group differences in the light of scan–rescan variability (Veraart et al., 2021) rather than r_{eff} differences within the splenium captured by our histology.

Figure S10.1d shows the statistical power as a function of the group size, assuming equal group sizes for subjects with ASD and healthy controls. A typical statistical power target of 0.9 could be reached for group sizes of 12 subjects at $\mathrm{SNR} \approx 27$, reflecting the conditions of our experimental protocol. However, increased $\mathrm{SNR} \geq 48$ would strongly reduce the group size requirements to 6 or fewer subjects per group.

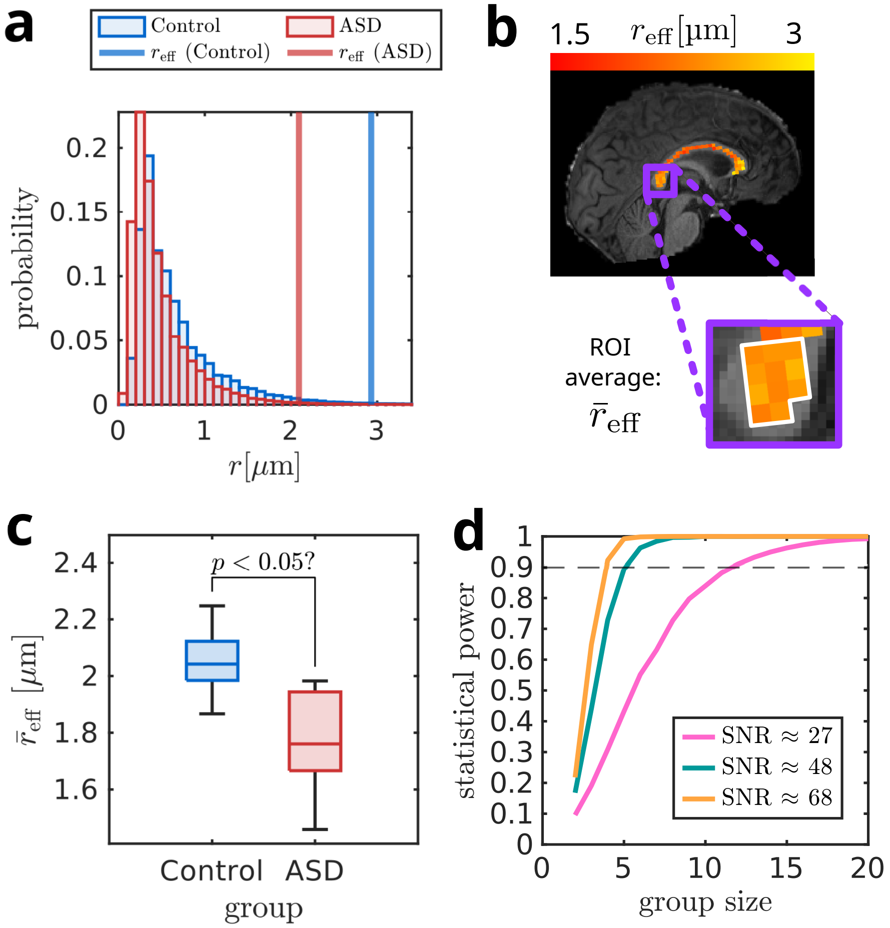

Figure S10.1: **Simulation of clinical application for r_{eff} mapping.** **(a)** Exemplary axon radius distributions in the splenium for subjects with autism spectrum disorder (ASD) and healthy controls, based on distributions from a splenium ROI in Figure 1a. ASD conditions were simulated by scaling down radii by $28.6\,\%$ (Wegiel et al., 2018), whereas distributions for healthy controls were used as is. **(b)** Illustration of subject-level mean r_{eff} in the splenium, denoted as $\bar{r}_{\mathrm{eff}}$. Values were computed over 11 voxels, reflecting the average number of splenium voxels in the mid-sagittal slice in our in-vivo dMRI subjects. **(c)** Illustration of one Monte Carlo iteration for statistical power estimation. We assessed group differences in $\bar{r}_{\mathrm{eff}}$ between healthy and ASD subjects with a t-test using a significance level $\alpha = 0.05$, with the null hypothesis being that the group means are equal. We then approximated the statistical power over 5000 Monte Carlo iterations as the fraction of tests that rejected the null hypothesis. **(d)** Statistical power as a function of the group size. Colors indicate optimal next-generation clinical scanner protocols for different SNR baseline levels determined in Figure 8c-e. The dashed line indicates a typical statistical power target value of 0.9. We assumed equal group sizes.

S11 Impact of D_0 variation

We assumed a fixed value for the axoplasmic diffusivity D_0 in the main manuscript. However, D_0 may vary across brain regions and individuals. To examine the impact of such variability on the correlation between in-vivo dMRI-based r_{eff} and histological values under various SNR conditions, we repeated the simulations for optimal next-generation clinical scanner protocols in Figure 8c–e, but with D_0 randomly varied across ROIs for signal generation, while using the fixed assumption ($D_0 = 0.35\,\mu\text{m}^2/\text{ms}$ ex-vivo and $D_0 = 2.07\,\mu\text{m}^2/\text{ms}$ in-vivo) for r_{eff} estimation. To enforce positive D_0, we sampled D_0 (for signal generation) from a lognormal distribution with the fixed value as mean μ and coefficient of variation $\text{CV} = 0.65$, reflecting the maximal intra-regional variability reported by Veraart et al. (2018) (intra-region variability of D_a for the "green" subject in the splenium in their Figure 8). The lognormal parameters were chosen accordingly as $\sigma_\ell^2 = \ln(1 + \text{CV}^2)$ and $\mu_\ell = \ln(\mu) - \frac{1}{2}\sigma_\ell^2$.

Figure S11.1 shows the comparison of simulated ex-vivo dMRI-based r_{eff} against histological values. This plot is the variability-augmented analog of Figure 5b. The correlation with variable D_0 is slightly weaker compared to the fixed-D_0 case ($R = 0.47$ versus $R = 0.55$, cf. Figure 5b), but the effect is rather mild.

Figure S11.2 shows the corresponding comparisons of simulated in-vivo dMRI-based r_{eff} against histological values at different SNR levels. These plots are the variability-augmented analog of Figure 8c-e. At $\text{SNR} \approx 27$ (see Figure S11.2a), R is slighty increased compared to the fixed-D_0 case ($R = 0.30$ versus $R = 0.29$, cf. Figure 8c), potentially reflecting an elevation of individual data points above the resolution limit for small r_{eff}. At higher SNR (see Figure S11.2b-c), R is reduced compared to the fixed-D_0 case ($R = 0.46$ versus $R = 0.49$ at SNR ≈ 48; $R = 0.55$ versus $R = 0.63$ at SNR ≈ 68; cf. Figure 8d-e). However, the impact is rather mild compared to the strong assumed variation of D_0.

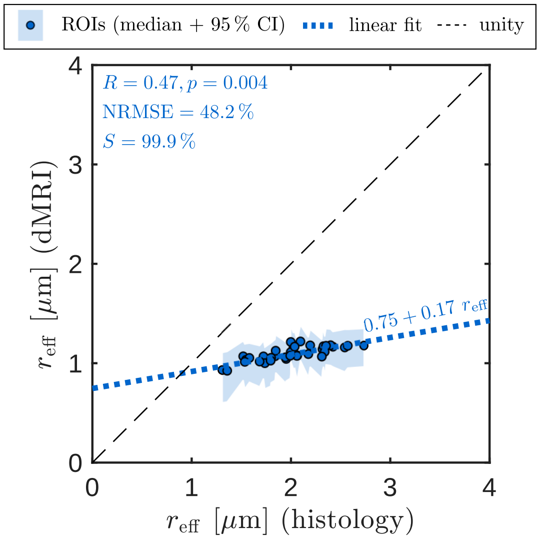

Figure S11.1: **Impact of D_0 variation on ex-vivo r_{eff} mapping.** Comparison of simulated r_{eff} with histological values for the experimental ex-vivo dMRI protocol. Here, dMRI signal simulations were conducted using axoplasmic diffusivities (D_0) randomly sampled from a lognormal distribution with variation estimated from literature values (Veraart et al., 2018). For r_{eff} estimation, we assumed fixed D_0, mimicking our approach for experimental dMRI. Markers represent histological ROIs in Figure 1a. The $95\,\%$ confidence intervals (shaded areas) were computed across 1000 noise realizations. The dashed line illustrates theoretical perfect agreement. Annotated metrics were computed over all ROIs, including Pearson's correlation coefficient (R) and the corresponding p-value, the normalized root-mean-square error (NRMSE), and the fitting success rate (S) (see Equations (6) to (9)).

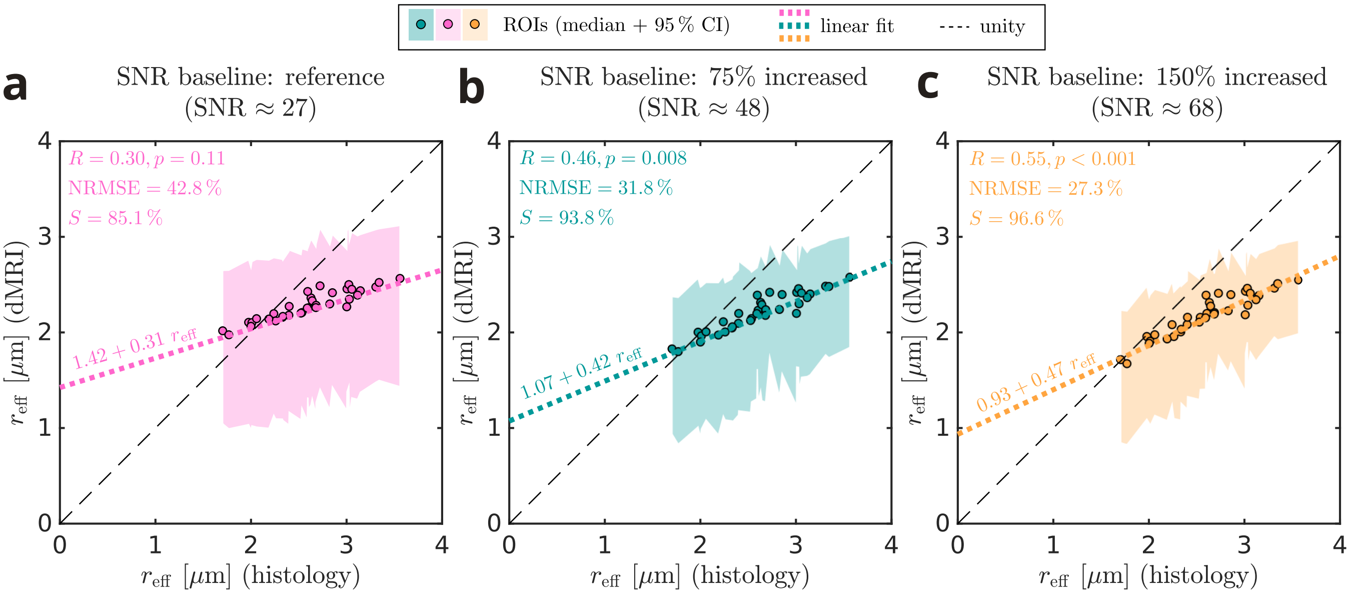

Figure S11.2: **Impact of D_0 variation on in-vivo r_{eff} mapping. (a-c)** Comparison of estimated r_{eff} with histological values for optimal next-generation clinical scanner protocols across baseline SNR levels. Here, dMRI signal simulations were conducted using axoplasmic diffusivities (D_0) randomly sampled from a lognormal distribution based on literature values (Veraart et al., 2018). For r_{eff} estimation, we assumed fixed D_0, mimicking our approach for experimental dMRI. Markers represent histological ROIs in Figure 1a. The $95\,\%$ confidence intervals (shaded areas) were computed across 1000 noise realizations. The dashed lines illustrate theoretical perfect agreement. The annotated metrics were computed over all ROIs, including Pearson's correlation coefficient (R) and the corresponding p-value, the normalized root-mean-square error (NRMSE), and the fitting success rate (S) (see Equations (6) to (9)).

S12 Histological radius approximations

Estimation of histological r_{eff} requires individual axon radii, which can be approximated from 2D cross-sections in different ways. In the main manuscript, we used the circular equivalent approximation; here, we additionally assess ellipse-based approximations using the fitted minor and major axes.

Figure S12.1 compares r_{eff} obtained with ellipse-based radius approximations against the circular equivalent approximation used in the main manuscript. r_{eff} estimates based on the ellipse minor axis closely matches the circular equivalent approximation (see Figure S12.1a), whereas the ellipse major axis strongly overestimates r_{eff} (see Figure S12.1b). This discrepancy likely reflects the susceptibility of the major-axis approximation to very elongated axons, which could be caused by non-orthogonal sectioning rather than true biological morphology.

Figure S12.2 repeats the comparison of dMRI-based r_{eff} against histological values in the main manuscript (see Figure 5a) using different radius approximations. dMRI-based r_{eff} align similarly well with histology-based r_{eff} when using the circular equivalent or ellipse minor axis approximation (see Figure S12.2a–b), consistent with the close agreement between these two histological approximations in Figure S12.1a. In contrast, when histological radii are defined by the ellipse major axis, dMRI-based r_{eff} show poor agreement (see Figure S12.2c), with the limited dynamic range of dMRI-based r_{eff} appearing even smaller relative to the large spread of ellipse major axis–based r_{eff}.

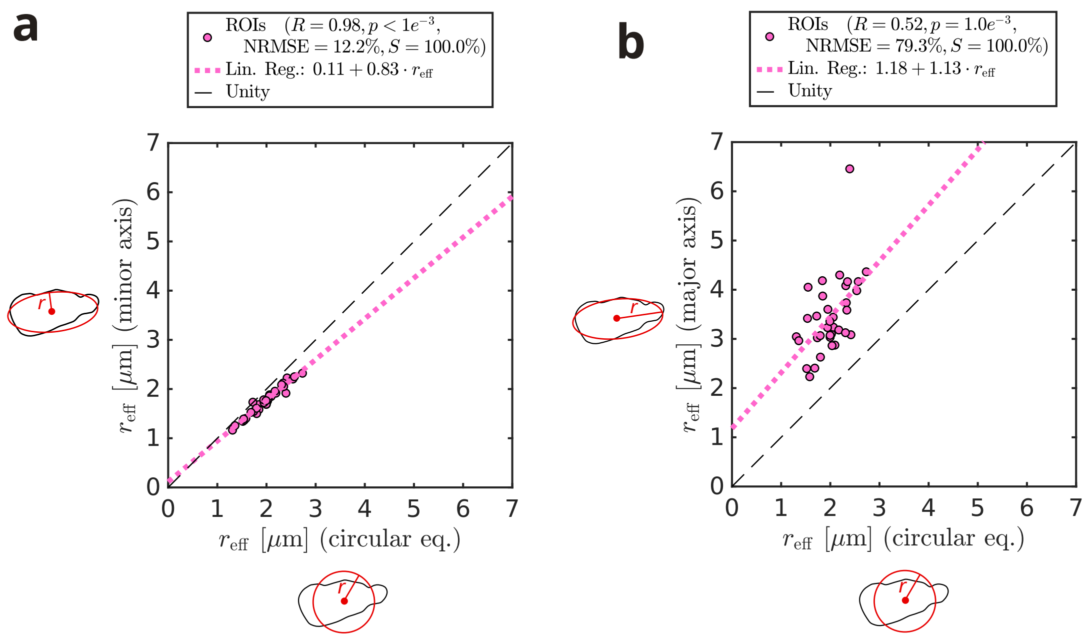

Figure S12.1: **Comparison of histological r_{eff} for various radius approximations. (a–b)** Comparison of r_{eff} using ellipse-based axon radius approximations against circular equivalent-based r_{eff}: **(a)** ellipse minor axis and **(b)** ellipse major axis. Markers represent histological ROIs in Figure 1a. The dashed lines illustrate theoretical perfect agreement. The legends provide metrics computed over all ROIs, including Pearson's correlation coefficient (R) and the corresponding p-value, the normalized root-mean-square error (NRMSE), and the fitting success rate (S) (see Equations (6) to (9)).

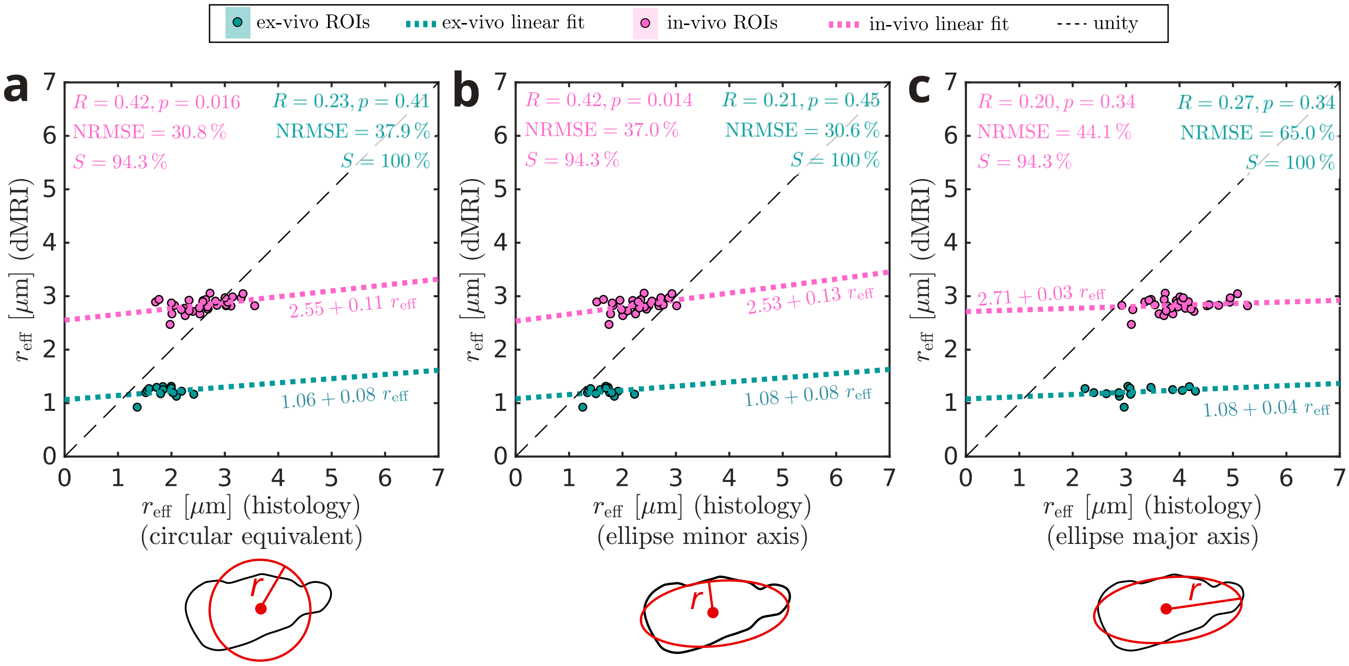

Figure S12.2: **Experimental validation of r_{eff} for various radius approximations. (a–c)** Quantitative comparisons of dMRI-based r_{eff} against histological r_{eff} using different radius approximations: **(a)** circular equivalent, **(b)** ellipse minor axis, and **(c)** ellipse major axis. Markers represent ROIs in Figure 1a, with colors indicating ex-vivo and in-vivo conditions. Ex-vivo markers include 15 ROIs of CC-01 (see Figure 2b,e), whereas in-vivo markers denote group-averaged r_{eff} values (see Section S5 for per-subject data). Dashed lines indicate perfect agreement. Annotated metrics were computed over all ROIs, including Pearson's correlation coefficient (R) with p-value, the normalized root-mean-square error (NRMSE), and the fitting success rate (S) (see Equations (6) to (9)). Panel (a) is replicated from Figure 5a for direct comparison across approximations.
